# Comparative Mitogenomic Analysis of Water Scavenger Beetles (Coleoptera: Hydrophiloidea) Provides Insights into Phylogeny and Adaptive Evolution

**DOI:** 10.3390/biology15070571

**Published:** 2026-04-02

**Authors:** Huan Wang, Han-Hui-Ying Lv, Yi-Yang Zhao, Shi-Yun Hu, Feng-Yi Gan, Yu-Xiang Wang, Ming-Long Yuan

**Affiliations:** 1 College of Grassland Science, Xinjiang Agricultural University, Urumqi 830052, China; 13993665785@163.com; 2 State Key Laboratory of Herbage Improvement and Grassland Agro-Ecosystems, College of Pastoral Agricultural Science and Technology, Lanzhou University, Lanzhou 730020, China; lvhhy2025@lzu.edu.cn (H.-H.-Y.L.); zhaoyy2025@lzu.edu.cn (Y.-Y.Z.); hushy20@lzu.edu.cn (S.-Y.H.); ganfy2023@lzu.edu.cn (F.-Y.G.)

**Keywords:** Qinghai-Tibetan Plateau, mitochondrial genomes, phylogeny, non-coding regions

## Abstract

Water scavenger beetles (Coleoptera: Hydrophiloidea) exhibit remarkable adaptations to both aquatic and terrestrial habitats and play irreplaceable ecological roles, yet their higher-level phylogeny and the molecular basis of their adaptive evolution remain unresolved. Here, we sequenced four complete mitogenomes of *C. unipunctatus* from the Qinghai–Tibetan Plateau (QTP) and conducted a comprehensive mitogenomic analysis by integrating these new data with 22 publicly available Hydrophiloidea mitogenomes, representing three families and six subfamilies. Our analyses revealed highly conserved mitogenomic architecture across Hydrophiloidea, with structural variation confined to non-coding regions and AT content correlating with both habitat type and phylogenetic lineage. Phylogenetic reconstructions robustly resolved the higher-level relationships: Helophoridae and Hydrochidae formed the sister group to Hydrophilidae, within which Hydrophilinae and Sphaeridiinae were strongly supported as monophyletic clades. We detected positive selection in the energy metabolism genes *cox3* and *nad5* along the ancestral branch of the terrestrial subfamily Sphaeridiinae, providing the first molecular evidence linking mitochondrial adaptation to the aquatic-to-terrestrial habitat shift. However, no positive selection signals were found in high-altitude *C. unipunctatus* populations, suggesting that adaptation to extreme environments operates through alternative mechanisms (e.g., regulatory or structural variation) rather than protein-coding evolution. This study establishes a robust phylogenetic framework for Hydrophiloidea and reveals the diverse molecular pathways underlying their adaptive evolution, from protein-coding adaptation during aquatic–terrestrial shifts to non-coding responses in extreme high-altitude environments.

## Simple Summary

Water scavenger beetles (Coleoptera: Hydrophiloidea) have remarkable aquatic–terrestrial adaptations and important ecological roles, yet their higher-level phylogeny and the molecular basis of adaptive evolution remain incompletely resolved. Here, we sequenced four complete mitochondrial genomes of *Cercyon unipunctatus* (Linnaeus, 1758) from the Qinghai–Tibetan Plateau and analyzed them together with 22 published Hydrophiloidea mitogenomes representing three families and six subfamilies. Mitogenomic architecture was highly conserved across Hydrophiloidea, with most structural variation confined to non-coding regions; moreover, AT content was associated with both habitat type and phylogenetic lineage. Phylogenetic reconstructions supported Helophoridae and Hydrochidae as the sister group to Hydrophilidae, within which Hydrophilinae and Sphaeridiinae were strongly recovered as monophyletic clades. We detected signals of positive selection in the energy-metabolism genes *cox3* and *nad5* along the ancestral branch of the terrestrial subfamily Sphaeridiinae, consistent with mitochondrial involvement in the aquatic-to-terrestrial habitat shift, whereas no such signals were observed in high-altitude *C. unipunctatus* populations. This study provides a mitogenome-based phylogeny and reveals distinct adaptive molecular routes.

## 1. Introduction

The superfamily Hydrophiloidea (Coleoptera: Staphyliniformia), commonly known as water scavenger beetles, is a species-rich lineage with a worldwide distribution [1]. This superfamily comprises six families and occupies a remarkable breadth of habitats, ranging from freshwater and semi-aquatic environments to fully terrestrial microhabitats [2,3]. This repeated association with both aquatic and terrestrial niches makes Hydrophiloidea an attractive system for investigating habitat shifts, trait evolution, and the ecological diversification of beetles. In many ecosystems, hydrophiloid beetles function as important decomposers, predators, and scavengers, thereby contributing to nutrient cycling and food-web dynamics [4].

The classification of Hydrophiloidea has traditionally relied on morphological characters [2,5]; however, higher-level relationships remain debated [6]. Notably, the phylogenetic placements of Helophoridae and Hydrochidae, as well as the relationships among subfamilies within Hydrophilidae, have not been well resolved [7,8]. Early molecular studies provided initial hypotheses for intergroup relationships, but limited marker sampling and restricted taxon coverage often failed to resolve deep nodes and, in some cases, amplified conflicts between morphology-based classifications and molecular inferences [9,10].

Mitochondrial genomes (mitogenomes) have increasingly been used to explore both phylogenetic relationships and adaptive evolution [11], particularly under extreme environmental stress [12]. Because mitochondria encode core components of oxidative phosphorylation, mitogenomes are frequently examined in the context of energetic constraints such as high-altitude hypoxia [13]. In vertebrates, evidence suggests that hypoxia-related adaptation may involve changes in regulatory regions, mitochondrial copy number, and mito–nuclear interactions, without necessarily requiring pervasive positive selection across protein-coding genes (PCGs) [14,15,16]. Comparable patterns have also been reported in invertebrates, where adaptive responses can involve non-sequence-dependent mechanisms or subtle shifts in selective regimes rather than widespread amino-acid replacements [17,18]. However, mitogenomic data for Hydrophiloidea remain relatively scarce [8], severely limiting our ability to gain deeper insights into the genomic evolutionary patterns of this group.

In this study, we present the first complete mitogenomes of four *C. unipunctatus* specimens collected from the QTP. By integrating complete mitogenomic data from 22 previously published Hydrophiloidea species retrieved from GenBank, we conducted a comprehensive comparative mitogenomics analysis across six subfamilies of Hydrophiloidea. The research objectives are as follows: (1) to characterize the structural features, nucleotide composition, and non-coding region characteristics of Hydrophiloidea mitogenomes; (2) to reconstruct a robust phylogenetic framework for the superfamily using multiple datasets and inference methods; and (3) to investigate mitochondrial adaptive evolution, specifically to test for positive selection linked to the aquatic-to-terrestrial habitat shift and to explore whether high-altitude environments have driven adaptive evolution in QTP populations. Our results not only refine the Hydrophiloidea phylogeny but also provide the first molecular evidence for mitochondrial PCG adaptation underlying the aquatic-to-terrestrial shift, while revealing the complexity of high-altitude adaptation beyond protein-coding evolution.

## 2. Materials and Methods

### 2.1. Sampling and DNA Extraction

Four newly sequenced specimens of *C. unipunctatus* were collected from alpine meadows in Qumalai County, Jiuzhi County, Zêkog County, and Zadoi County, Qinghai Province, China, in August 2022 and July 2023, respectively (Table S1). Field-collected specimens were initially preserved in absolute ethanol and subsequently transported to Lanzhou University, where they were stored long-term at −80 °C. Genomic DNA was extracted from adult hind leg using the CTAB method, respectively [19]. The concentration and purity of DNA were evaluated using a NanoDrop ND-1000 spectrophotometer (Thermo Fisher Scientific, Waltham, MA, USA), and DNA integrity was verified by 1.2% agarose (Sangon Biotech, Shanghai, China) gel electrophoresis.

### 2.2. Mitogenome Sequencing, Assembly, and Annotation

Paired-end sequencing libraries (2 × 150 bp) were constructed by Wuhan Benagen Tech Solutions Company Limited and sequenced on the Illumina NovaSeq 6000 platform (Wuhan, China). The methods used for quality control of raw sequencing reads, assembly and alignment identification of high-quality clean reads, as well as annotation of the complete circular mitogenomes, were consistent with those described by Wang et al. [20]. The boundaries of PCGs were manually verified by predicting open reading frames (ORFs) and aligning sequences against homologous mitogenomes of closely related Hydrophiloidea beetle species. Prediction of the secondary structures of transfer RNA (tRNA) genes, annotation of the two ribosomal RNA genes (*rrnL* and *rrnS*) based on sequence homology, and construction of circular mitogenome maps were all performed following the methods described by Wang et al. [20].

### 2.3. Comparative Mitogenomic Analysis of Hydrophiloidea

A comparative mitogenomic analysis was conducted on 26 mitogenomes representing the superfamily Hydrophiloidea. This dataset included our newly sequenced mitogenomes plus 22 additional Hydrophiloidea mitogenomes retrieved from the GenBank database [8,21,22] (Table S2). Based on relevant references, the habitat (aquatic, semi-aquatic, or terrestrial) was compiled for each species [23,24,25,26,27,28,29,30,31] (Table S3).

Nucleotide composition, AT-skew [(A − T)/(A + T)], and GC-skew [(G − C)/(G + C)] [32] were calculated for the entire mitogenome, individual genes, and distinct codon positions using MEGA v12.1.0 [33]. Relative synonymous codon usage (RSCU) values for the 13 PCGs were also calculated with MEGA v12.1.0 [33]. The effective number of codons (ENC) and codon bias index (CBI) were determined using DnaSP v6.12.3 [34], and their correlations with overall GC content and GC content at the third codon position (GC3) were subsequently analyzed.

To evaluate interspecific genetic divergence, nucleotide diversity (*Pi*) for each PCG across all species was estimated via a sliding-window approach (window length: 100 bp; step size: 25 bp) using DnaSP v6.12.3 [34]. Subsequently, we analyzed seven gene fragments (*atp8*, *cox1*, *cox3*, *nad3*, *nad5*, *trnA* and *trnR*) amplified from individuals of three distinct populations. Sequence conservation among the three high-altitude populations of *C. unipunctatus* was assessed by comparing the number of variable sites (*S*), *Pi*, and haplotype diversity (*Hd*) values across populations. The non-synonymous mutation rate (*Ka*) and synonymous mutation rate (*Ks*) for each PCG were calculated using MEGA v12.1.0 [33]. Selective pressure acting on individual PCGs was assessed using the branch-site model implemented in the codeml program of PAML v4.10.9 [35], with Sphaeridiinae or *C. unipunctatus* (high-altitude species) designated as the foreground branch. Positively selected sites were defined as those with *ω* > 1 and a significant likelihood ratio test (LRT) result (*p* < 0.05) [35]. The Bayes empirical Bayes (BEB) method was used to calculate posterior probabilities of site classes, enabling the identification of specific codon positions subjected to positive selection (*ω* > 1) [36].

Sequence alignment of PCGs and non-coding regions was performed using MEGA v12.1.0 [33]. Tandem repeats within the control region (CR) were identified using Tandem Repeats Finder [37] under default parameters. Based on these repeat identifications, a schematic diagram illustrating the repeat structure and conserved regions was generated for visual comparative analysis.

### 2.4. Phylogenetic Analysis

By combining these newly sequenced mitogenomes with 22 published sequences retrieved from GenBank, we constructed three datasets for phylogenetic inference: (1) P123: concatenated nucleotide sequences of all 13 PCGs (11,130 bp); (2) P123AA: concatenated amino acid sequences of the 13 PCGs (3710 amino acids); (3) P123R: concatenated P123 nucleotides plus the two rRNA genes (*rrnL* and *rrnS*) (13,293 bp). Each PCG was aligned separately at the codon level using MAFFT v7.505 [38], while rRNA genes were aligned using the Q-INS-i algorithm in MAFFT, which considers secondary structure. Two species from the Histeridae family, *Euspilotus scissus* (GU176344) and *Margarinotus merdarius* (NC_028603), were used as outgroups (Table S2).

We used DAMBE v7.0.35 [39] to test substitution saturation and no significant saturation was found (Table S4). The best-fit partitioning schemes and corresponding substitution models for each dataset were selected using IQ-TREE v2.2.0 [40] via the ModelFinder option [41], which minimizes the Bayesian Information Criterion (BIC). The best schemes and evolutionary models are provided in Table S5.

We performed phylogenetic analyses using the Maximum Likelihood (ML) method and Bayesian Inference (BI). The analytical procedures for both ML and BI were performed following the methods described by Wang et al. [20]. Each phylogenetic tree were visualized and annotated using FigTree v1.4.5 [42]. We used IQ-TREE v2.2.0 [40] to perform topology tests for three family-level relationships within Hydrophiloidea. We performed SH (Shimodaira–Hasegawa), KH (Kishino–Hasegawa), ELW (expected likelihood weight), and AU (approximately unbiased) [43,44,45,46] tests for each of the three datasets (P123, P123AA, and P123R) with 1000 replicates. All tests were performed with 1000 resamplings using the RELL method.

## 3. Results

### 3.1. Mitogenomic Features of High-Altitude Cercyon Unipunctatus Populations

**Figure 1:**
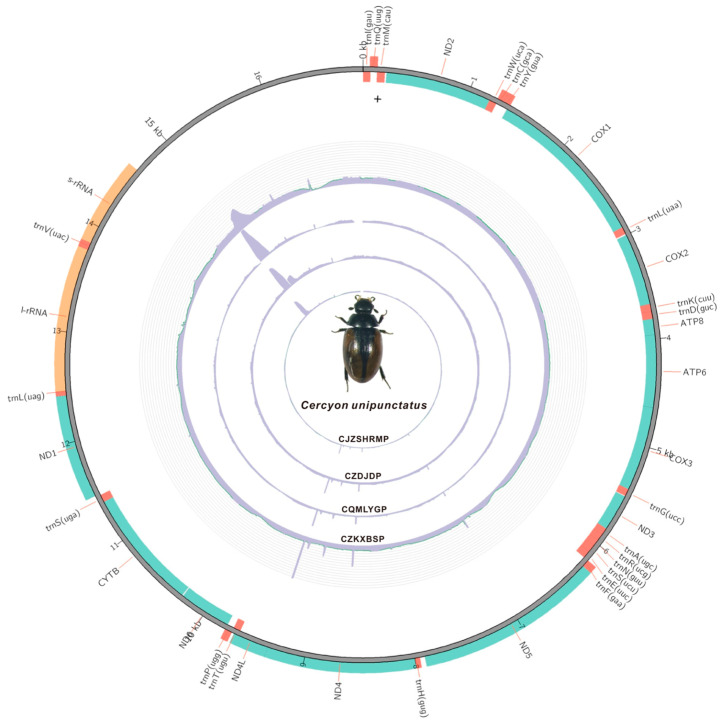
Circular map of the four *Cercyon unipunctatus* mitogenomes. The map depicts the organization of 37 mitochondrial genes: 13 protein-coding genes (PCGs, green), 22 transfer RNA genes (tRNAs, orange), and two ribosomal RNA genes (rRNAs, yellow). The major non-coding CR is shown in gray. Genes on the outer strand are transcribed clockwise. From outer to inner, the four concentric circles represent the mitogenomes of four *Cercyon unipunctatus* collected from different high altitudes: CZKXBSP, CQMLYGP, CZDJDP, and CJZSHRMP.

**Figure 2:**
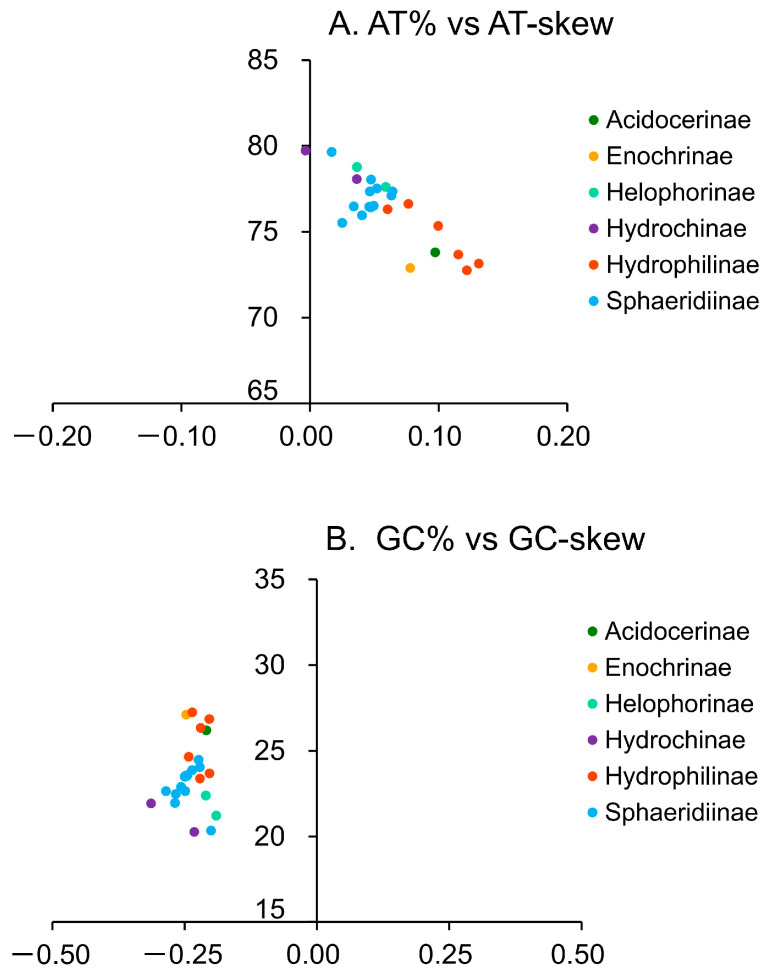
Nucleotide composition skews in Hydrophiloidea mitogenomes. (**A**) AT-skew plotted against overall AT content; (**B**) GC-skew plotted against overall GC content. Values were calculated for the entire major strand (J-strand) of each mitogenome. Colors represent different subfamilies. The four newly sequenced individuals belong to Sphaeridiinae.

We obtained the complete mitogenomes of four geographically distinct individuals of *C. unipunctatus* from the QTP, with the size ranging from 16,807 bp to 16,938 bp, with identical coding region lengths (Figure 1 and Figure S1). Among the 13 PCGs, the AT content of the four newly sequenced individuals did not exhibit extreme values relative to other species (Figure S2). The AT content of the entire mitogenomes across the four individuals varied from 75.96% to 76.46% (Figure 2A), whilst the GC content ranged from 23.54% to 24.04% (Figure 2B), indicating a marked AT skew (Figure 2). The AT content of the PCGs was 75.22–75.24%, displaying minimal variation among individuals (Figure S2), whereas the CR exhibited the highest AT content (79.38–79.74%). We compared the mitogenomes of four individuals of the same species collected from high-altitude regions and detected an extremely low overall level of genetic variation. Among the 13 PCGs, nucleotide sequence identity reached 99.94% among individuals, with a corresponding amino acid sequence identity of 99.89%. Similarly, the 22 tRNA genes and two rRNA genes exhibited minimal genetic divergence. Codon usage analysis indicated that newly sequenced species exhibited a greater preference for AT-terminating codons than other Hydrophiloidea mitogenomes (Figure S3).

**Figure 3:**
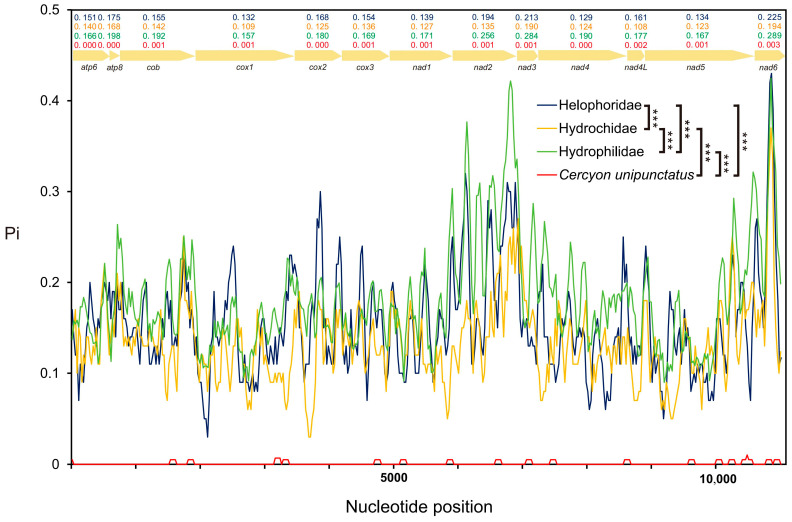
Nucleotide diversity (*Pi*) of mitochondrial protein-coding genes. Sliding window analysis (window: 100 bp, step size: 25 bp) was performed to compare sequence polymorphism across four groups: newly sequenced *Cercyon unipunctatus* populations (red line), Helophoridae (blue line), Hydrochidae (yellow line), and Hydrophilidae (green line). Statistical comparisons between groups are indicated (*** *p* < 0.001; independent samples *t*-test).

Results verifying intraspecific sequence conservation revealed that conserved fragments (*atp8*, *cox3*, *trnA*, and *trnR*) exhibited almost no polymorphic sites across all population samples, whereas a small number of stable variant sites were identified in *nad5* (Table S6). Specifically, a total of 249 variable sites were identified in *cox1*, among which the higher-altitude population contained up to 202 variable sites; in contrast, no variable sites were detected in *atp8* (CQMLYGP population), *cox3* gene (CJZSHRMP population), or *nad3* (CJZSHRMP population). Although *Pi* and *Hd* of high-altitude populations were slightly higher than those of relatively low-altitude populations across the three groups (Table S6), overall levels remained relatively low. *Pi* of the 13 PCGs approached zero in the four newly sequenced species (Figure 3). Notably, branch-site model analysis of the high-altitude lineage (*C. unipunctatus*) revealed no significant signals of positive selection in any of the 13 PCGs.

**Figure 4:**
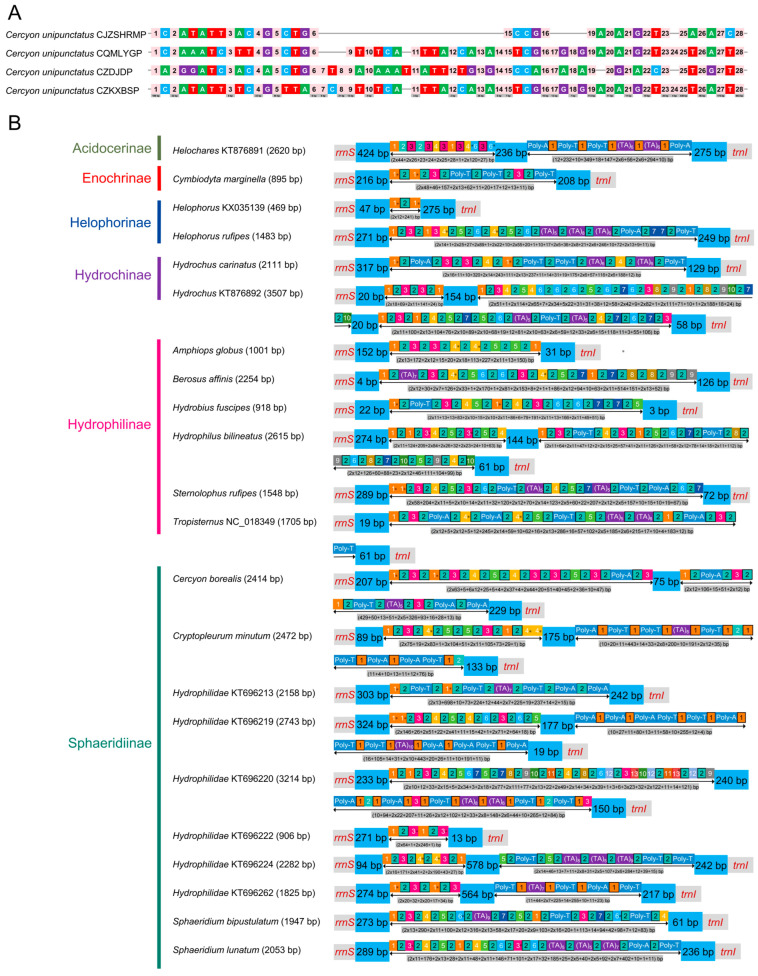
Structural organization of the mitochondrial control region. (**A**) Schematic representation of conserved structural elements in the *C. unipunctatus* control regions. Pink numbered squares indicate independent conserved regions (>2 bp). Gray bar indicates scale. Green, red, blue, and purple represent A, T, C, and G bases, respectively. (**B**) Comparative organization of the mitochondrial control region across Hydrophiloidea. The region between *rrnS* and *trnI* is shown for representative species. Annotated features include: light blue blocks (non-repetitive spacers), colored numbered squares (unique tandem repeat units), bordered squares (inter-repeat spacers), purple blocks (TA motifs), blue blocks (poly-A/poly-T/AT-rich stretches). Colored squares marked with asterisks (*) correspond to the longest repetitive sequences detailed in Table S9.

Distinct conserved motifs were identified in the CR sequences of different individuals. The full length of the CRs ranged from 2187 bp to 2318 bp, with a sequence identity of 93.4%. Length variation was primarily driven by insertions and deletions (InDels) in the central repetitive region, while sequences at both termini were identical, a pattern that was particularly prominent in the CJZSHRMP individual. Segmental analysis of CR sequences identified 28 conserved regions longer than 2 bp, with the longest spanning 943 bp (Figure 4A).

### 3.2. Comparative Mitogenomics of Hydrophiloidea: Architecture, Composition, and Evolution

All Hydrophiloidea mitogenomes contained the typical set of 37 genes: 13 PCGs, 2 rRNAs, and 22 tRNAs, with no gene rearrangement (Table S7). Mitogenome lengths varied substantially, ranging from 15,139 bp in *Helophorus* to 18,299 bp in *Hydrochus*; this size variation was primarily attributed to length polymorphism in the CR (Figure S1).

**Figure 5:**
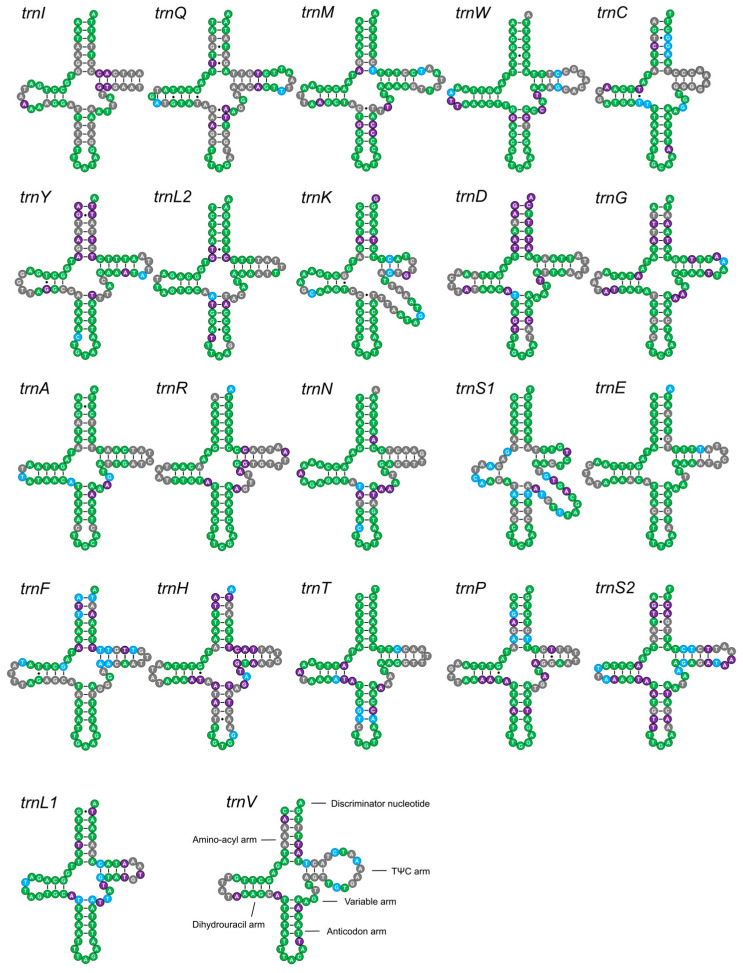
Predicted secondary structures of the 22 tRNA genes across Hydrophiloidea mitogenomes. tRNAs are displayed in their genomic order, starting with *trnI*. Nucleotide conservation is color-coded: green (conserved across all Hydrophiloidea), blue (conserved within Hydrophilidae), purple (conserved within Sphaeridiinae), and gray (not conserved). Watson–Crick pairings are indicated by bars and G-U wobble pairs by dots.

The secondary structures of the 22 tRNA genes in Hydrophiloidea were highly conserved across Hydrophiloidea, all conforming to the canonical cloverleaf structure (Figure 5). The sequence identity of tRNA genes was 62.25% at the family level, reaching up to 92.73% in some subfamilies, most notably Hydrochinae (Figure 5). The lengths of the *rrnS* and *rrnL* were 769–787 bp and 1280–1302 bp, respectively, with AT contents exceeding 82% for both. Sequence conservation was highest within individual subfamilies, particularly Hydrochinae.

**Figure 6:**
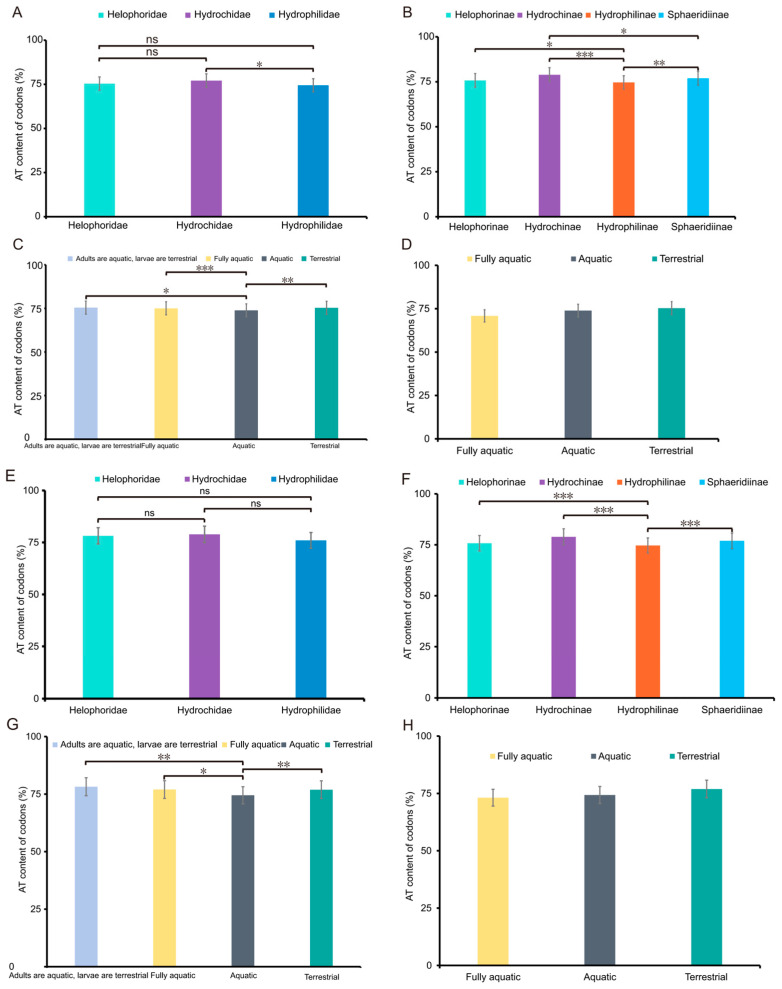
Variation in mitogenomic AT content across Hydrophiloidea taxonomic levels and habitat types. (**A**) AT content variation among three families; (**B**) AT content variation among four subfamilies; (**C**) AT content comparison among Hydrophiloidea species with different habitat types; (**D**) AT content comparison among Hydrophilidae species with different habitat types; (**E**) AT content variation in PCGs among three families; (**F**) AT content variation in PCGs among four subfamilies; (**G**) AT content comparison among Hydrophiloidea species with different habitats; (**H**) AT content comparison among Hydrophilidae species with different habitats. Statistical significance: * *p* < 0.05, ** *p* < 0.01, *** *p* < 0.001; ns, not significant (independent samples *t*-test).

All mitogenomes exhibited a strong AT bias, with total AT content ranging from 77.62% in *Amphiops globus* to 81.24% in *Sternolophus rufipes* (Figure 2). The total AT content of Hydrophilidae was significantly lower than that of Hydrochidae (Figure 6A). Within Hydrophilidae, the total AT content of Hydrophilinae was significantly lower than that of Helophorinae, Hydrochinae, and Sphaeridiinae (Figure 6B).

Notably, the third codon position had the highest average AT content (87.71%), significantly greater than that of the first and second codon positions (Figure S2). No significant differences in AT content were observed across the three families at the PCG level (Figure 6E). However, significant variation existed among subfamilies, with Hydrophilinae showing significantly lower AT content than the other three subfamilies (Figure 6F).

The *Ka*/*Ks* value for each of the 13 PCGs was below 1 (range: 0.05–0.41), indicating that these genes are under strong purifying selection. *Nad6* had the highest evolutionary rate, while *cox1* was the most conserved (Figure S4A). Furthermore, *Ka*/*Ks* values of the PCGs showed a significant negative correlation with GC content (R^2^ = 0.71, *p* < 0.001; Figure S4B). *Ka*/*Ks* ratios varied across different families and subfamilies within Hydrophiloidea (Figure S5). Within Hydrochidae, *atp8* exhibited the highest evolutionary rate, whereas the *nad6* gene showed the highest rate in Helophoridae and Hydrophilidae (Figure S5A). Except for Hydrochinae, *nad6* displayed the highest evolutionary rate across all other subfamilies within Hydrophiloidea (Figure S5B). *Pi* of the 13 PCGs varied significantly among families. The *Pi* value of Hydrophilidae was significantly higher than that of the other three families, with the *nad6* gene exhibiting the highest polymorphism (*Pi* = 0.289), whereas the lowest *Pi* value (*Pi* = 0.134) was observed in Helophoridae (Figure 3).

**Figure 7:**
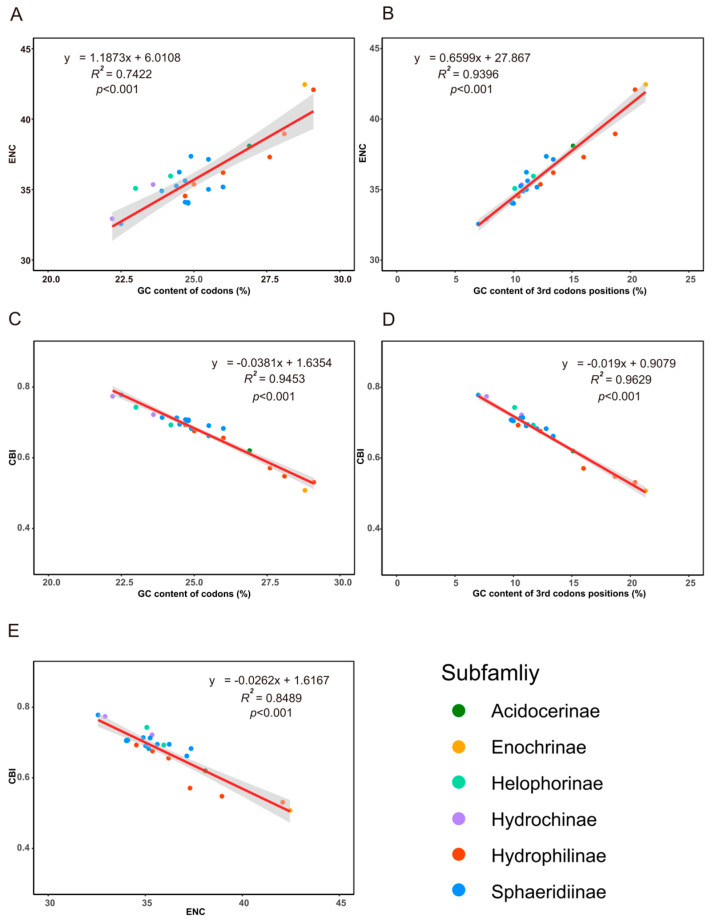
Codon usage bias in Hydrophiloidea mitogenomes. Correlations between (**A**) effective number of codons (ENC) and overall GC content (GC%); (**B**) ENC and third-position GC content (GC3%); (**C**) codon bias index (CBI) and GC%; (**D**) CBI and GC3%; (**E**) ENC and CBI. Shaded areas indicate 95% confidence intervals. Each point represents one species, with colors denoting subfamilies.

Codon usage analysis revealed a preference for AT-terminating codons in Hydrophiloidea mitogenomes (Figure S3B; Table S8). The ENC showed a strong positive correlation with both total GC content (GC%) and GC content at the third codon position (GC3%), whereas the CBI exhibited a negative correlation with these two metrics (Figure 7). All species plotted below the expected ENC curve, indicating that codon usage patterns may be influenced by factors other than mutation bias (Figure S6).

The CR, located between *rrnS* and *trnI*, was the primary driver of mitogenome size variation and contained AT-rich regions and repetitive sequences. Its length varied substantially, ranging from 469 bp in *Helophorus* to 3507 bp in *Hydrochus* (Figure 4B). Notably, no species exhibited distinct AT-rich characteristics in the CR (Figure 4B). *Helophorus* contained only one repeat unit, resulting in the shortest CR among all species (469 bp) (Table S9). Species with longer CRs exhibited more complex structures and greater diversity in repeat unit composition (Figure 4B). Furthermore, a short, highly conserved intergenic region (17–42 bp) was presented between *trnS2* and *nad1* across all species. This region showed 100% sequence conservation within Hydrochinae (Figure S7).

### 3.3. Mitochondrial Adaptation to the Aquatic–Terrestrial Habitat Shift

Across the entire Hydrophiloidea, aquatic species exhibited significantly lower total AT content than both semi-aquatic species (with aquatic adults and terrestrial larvae) and fully terrestrial species (Figure 6C). Conversely, within Hydrophilidae, terrestrial species (Sphaeridiinae) showed higher AT content than aquatic species (Figure 6D). Consistent with this, the AT content of PCGs in aquatic species displayed significantly lower total AT content than both semi-aquatic and terrestrial species (Figure 6G). Terrestrial species within Hydrophilidae exhibited higher AT content than aquatic species (Figure 6H).

*Ka*/*Ks* analysis revealed that fully aquatic species tended to exhibit the highest values across multiple genes, most notably *atp8*, *nad1*, *nad2*, *nad3*, *nad4*, *nad5*, and *nad6*. For most genes, differences in *Ka*/*Ks* values between aquatic and terrestrial species were minimal; however, terrestrial species displayed higher *Ka*/*Ks* values than aquatic species for *atp8*, *nad1*, *nad2*, and *nad4*. Semi-aquatic species also showed elevated *Ka*/*Ks* values in some genes: specifically, *cox2*, *cox3*, and *nad4L* exhibited higher values than those in the other three groups (Figure S8), suggesting that this life-history transition may be associated with distinct selective pressure patterns at specific mitochondrial genes or sites.

However, significant positive selection was detected in *cox3* and *nad5* along the ancestral branch of the terrestrial subfamily Sphaeridiinae. A total of four positively selected sites were detected in *cox3* and eight in *nad5*, all of which were statistically significant (BEB ≥ 0.95) (Table 1).

### 3.4. Phylogenomic Relationships Within Hydrophiloidea

**Figure 8:**
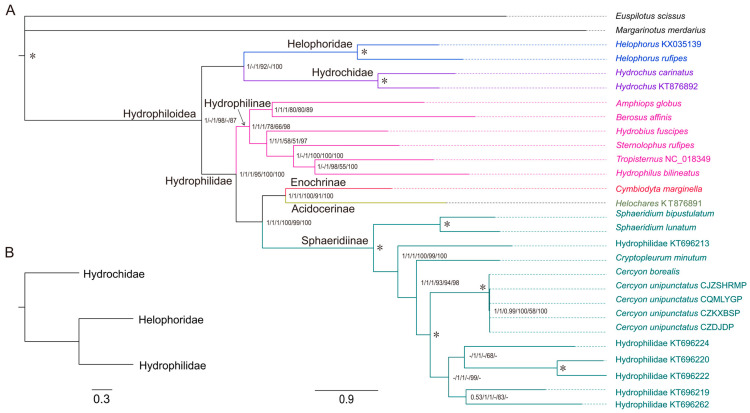
Phylogenetic relationships within Hydrophiloidea inferred from mitogenome sequences. (**A**) Phylogenetic tree reconstructed using two datasets (P123 and P123R) and two methods (BI and ML). Numbers from left to right are Bayesian posterior probabilities and maximum likelihood bootstrap values for each of the three datasets. Asterisk (*) indicates PP = 1.0 and BS = 100. (**B**) Alternative phylogenetic relationship among the three families, reconstructed using the P123AA dataset with two methods (BI and ML).

Phylogenetic relationships were constructed using three datasets (P123, P123AA, P123R) with ML and BI methods, generating six phylogenetic trees representing two distinct family-level topologies (Figure 8). One is the phylogenetic tree reconstructed using two datasets (P123 and P123R) and two methods (BI and ML) (Figure 8A). The other is the alternative phylogenetic relationship among the three families reconstructed based on the P123AA dataset with the two aforementioned methods (Figure 8B). Within the phylogenetic tree, while support values for most other nodes are generally high, this topological configuration was directly attributable to the unstable phylogenetic positions of *Helophorus* KX035139 and *Helophorus rufipes*. Across all datasets except P123AA, a strongly supported sister-group relationship between Helophoridae and Hydrochidae was recovered (PP = 1; Figure 8A). These two families form the clade and sister to Hydrophilidae (PP = 1). Topologies tests further validated this family-level relationship (Table S10). In contrast, dataset P123AA using two methods (BI and ML) recovered Helophoridae as the sister group to Hydrophilidae (Figure 8B).

All phylogenetic trees strongly support the monophyly of the three focal families, with high supporting values (Figure 8A). Within Hydrophilidae, two major clades were recovered: Hydrophilinae (fully aquatic) formed a highly supported monophyletic clade, and a second clade comprising Sphaeridiinae + (Enochrinae + Acidocerinae) was also recovered. Sphaeridiinae, encompassing *Sphaeridium* and *Cercyon*, with all constituent species exhibiting terrestrial habits, formed a maximally supported monophyletic clade (PP = 1.0), consistent with the independent evolution of terrestrial adaptations in this subfamily.

## 4. Discussion

### 4.1. A Robust Mitogenomic Phylogeny of Hydrophiloidea

This study provides molecular evidence for the monophyly of Helophoridae, Hydrochidae, and Hydrophilidae, consistent with previous phylogenetic studies of Hydrophiloidea based on transcriptomic data and multiple gene fragments [7,47]. A key finding of our study was the well-supported sister-group relationship between Helophoridae and Hydrochidae, which was further confirmed by topology tests. This relationship not only clarified their phylogenetic position but also had significant implications for understanding the evolutionary origins of aquatic adaptation within Hydrophiloidea [6,7]. Previous molecular studies [48,49] suggested a closer relationship between Hydrochidae and Hydrophilidae, a discrepancy likely attributed to limitations in genetic markers and insufficient taxa sampling. By using whole mitogenomic data, the present resolved this phylogenetic ambiguity, confirming that Helophoridae + Hydrochidae formed the sister group to Hydrophilidae, a finding fully consistent with earlier morphological studies [50] and recent genomic evidence [6].

Within Hydrophilidae, our results for the two major clades [Hydrophilinae + ((Enochrinae + Acidocerinae) + Sphaeridiinae)] aligned with the subfamily-level classification system proposed by Fikáček et al. (2019) [7,24]. Notably, the strong monophyly of Hydrophilinae and Sphaeridiinae supported earlier morphological [51] and molecular studies [7].

### 4.2. Mitochondrial Adaptation to the Aquatic-to-Terrestrial Habitat Shift

A central finding of this study is the signature of positive selection in two key mitochondrial energy metabolism genes, *cox3* and *nad5*, along the ancestral branch of the terrestrial subfamily Sphaeridiinae. This result directly addressed our objective of investigating mitochondrial adaptation associated with the aquatic-to-terrestrial transition. The positively selected sites in *cox3* (cytochrome c oxidase subunit III) are enriched in the transmembrane domain and regions associated with the proton channel, whereas those in *nad5* (NADH dehydrogenase subunit V) localize to regions adjacent to the proton pump or the ubiquinone-binding domain [52,53]. As core components of oxidative phosphorylation (OXPHOS) complex IV and I, respectively, *cox3* and *nad5* collectively determine the efficiency of the electron transport chain and baseline energy output [54,55].

We proposed that natural selection likely optimized the basal energy metabolism system of Sphaeridiinae ancestors to meet the heightened demands of terrestrial life. Enhanced ATP synthesis efficiency supports energetically costly activities such as locomotion, reproduction, and osmoregulation. Concurrently, these adaptive changes may improve mitochondrial tolerance to oxidative stress induced by higher atmospheric oxygen levels, mitigating cellular damage from reactive oxygen species (ROS) [56]. This pattern was consistent with observations in other taxa transitioning between aquatic and terrestrial habitats, where mitochondrial genes undergo selection to adapt to oxidative environments [57,58].

Importantly, our findings provide molecular evidence complementing morphological adaptation hypotheses, such as the evolution of piercing–sucking mouthparts, thereby contributing to a broader narrative of morphology–metabolism coevolution. The functional implementation of specialized morphological traits depends critically on efficient energy supply systems [59,60]. The positive selection we detected in *cox3* and *nad5* likely provided the metabolic foundation for muscle-driven behaviors and physiological processes associated with terrestrial foraging. This adaptive logic appears conserved across piercing–sucking insect lineages [61,62]. Future functional assays (e.g., measuring enzyme activity or ATP production in variants carrying these positively selected sites) would provide direct experimental validation of their adaptive significance.

### 4.3. High-Altitude Adaptation in Cercyon Unipunctatus: Beyond Protein Sequence Evolution

In contrast to the clear positive selection signal associated with the aquatic-to-terrestrial transition, our analysis detected no evidence of positive selection in mitochondrial PCGs from high-altitude *C. unipunctatus* populations. This negative result, rather than diminishing the value of adaptive evolution research, reveals deeper complexities in the mechanisms underlying high-altitude adaptation [63,64]. The extreme environment of the QTP, characterized by hypoxia, low temperatures, and intense ultraviolet radiation, undoubtedly exerts strong selective pressures on energy metabolism, membrane stability, and genetic integrity [65,66]. However, our findings suggest that adaptation to these challenges does not necessarily require amino acid replacements in core mitochondrial proteins.

We propose several non-mutually exclusive mechanisms that may explain the observed pattern. First, regulatory adaptation, variation in gene expression levels, timing, or tissue specificity, has emerged as a key mechanism in high-altitude adaptation [58,67], particularly when protein sequences remain conserved. As an important component of the regulatory region, variations in the CR participate in high-altitude hypoxia adaptation by regulating gene expression dynamics. Our observation of high protein-coding sequence conservation alongside significant variation in the CR provided direct empirical evidence supporting this hypothesis. Regulatory regions modulate gene expression dynamics without altering amino acid composition [68]; in high-altitude populations, variation in these regions could restructure gene regulatory networks to counter hypoxic stress and remodel energy metabolism [69].

Second, structural variation, particularly in the mitochondrial CR, may contribute to adaptation without modifying protein sequences. The CR governs mitochondrial DNA replication and transcription, and its high variability in high-altitude-adapted species [68] can modulate energy production by altering replication or transcription efficiency, thereby enhancing hypoxia tolerance [70]. Copy number variations represent another structural mechanism: duplication of genes involved in oxygen transport or DNA repair can increase expression levels in a dose-dependent manner, conferring adaptive advantages [71].

Third, nuclear-encoded mitochondrial genes may play a predominant role. As mitochondria are the primary source of cellular energy production, high-altitude adaptation is likely underpinned by nuclear genes that regulate mitochondrial biogenesis, autophagy, antioxidant defense, and metabolite transport. Adaptive modifications in these nuclear regulators can systematically reshape mitochondrial function without altering the protein-coding blueprint of the mitogenome itself [72,73].

Finally, preadaptation and neutral processes cannot be discounted. Genetic variation conferring tolerance to hypoxia and cold may have been present in ancestral lowland populations before colonization of high-altitude habitats [74,75]; such preexisting variation would be fixed during subsequent selection without requiring de novo positive selection events. Additionally, neutral genetic drift can drive the fixation of slightly beneficial variants in small founding populations with limited gene flow, expanding the genetic repertoire available for adaptation [76,77].

Collectively, these considerations highlight that high-altitude adaptation in *C. unipunctatus* likely involves a multi-layered response in which regulatory, structural, and nuclear-encoded mechanisms supersede protein sequence evolution in core mitochondrial genes. This interpretation aligns with growing recognition that adaptive evolution operates through diverse molecular pathways, and that the absence of positive selection in protein-coding sequences does not equate to the absence of adaptation [15].

## 5. Conclusions

This study presents the first complete mitogenomes of *C. unipunctatus* from the QTP, revealing highly conserved genomic architecture across Hydrophiloidea with the CR as the primary source of structural variation. Phylogenomic analyses robustly resolved the sister-group relationship between Helophoridae and Hydrochidae, establishing a well-supported framework for the superfamily. Critically, we detected significant positive selection in the energy metabolism genes *cox3* and *nad5* along the ancestral branch of the terrestrial subfamily Sphaeridiinae, providing the first molecular evidence for mitochondrial adaptation during the aquatic-to-terrestrial habitat shift. In contrast, no positive selection signals were found in QTP populations of *C. unipunctatus*, suggesting that adaptation to extreme environments likely operates through non-sequence-based mechanisms. These findings establish a robust evolutionary basis for understanding ecological transitions in this diverse beetle group and offer broader insights into the diverse molecular pathways through which organisms adapt to new environments.

## Figures and Tables

**Table 1:** Branch-site model identific Volume 1: Morphology and Systematics. Archostemate lineage. 2Δ*L*: twice the log-likelihood difference; BEB: Bayes empirical Bayes; *: posterior probability (PP) > 0.95; **: PP > 0.99.

Gene	Model	Likelihood (*L*)	Site Class	0	1	2a	2b	2Δ*L*	Bayesian Empirical Bayesian (BEB) Analysis Results
*cox3*	Model A (Null)	−6664.031448	proportion	0.9194	0.0071	0.0729	0.0006		
			background *ω*	0.0129	1.0000	0.0129	1.0000		
			foreground *ω*	0.0129	1.0000	1.0000	1.0000		
	Model A (Alternative)	−6659.904726	proportion	0.9582	0.0074	0.0341	0.0003		
			background *ω*	0.0129	1.0000	0.0129	1.0000		
			foreground *ω*	0.0129	1.0000	999.0000	999.0000	8.25	38F 0.997 **; 63S 0.953 *; 217A 0.969 *; 230S 0.999 **
*nad5*	Model A (Null)	−16,344.247426	proportion	0.8824	0.0435	0.0706	0.0035		
			background *ω*	0.0164	1.0000	0.0164	1.0000		
			foreground *ω*	0.0164	1.0000	1.0000	1.0000		
	Model A (Alternative)	−16,340.160649	proportion	0.8928	0.0445	0.0598	0.0030		
			background *ω*	0.0165	1.0000	0.0165	1.0000		
			foreground *ω*	0.0165	1.0000	6.8798	6.8798	8.17	45S 0.988 *; 46S 0.996 **; 93S 0.981 *; 424G 0.990 **; 467W 0.971 *; 476S 0.998 **; 494S 0.977 *; 516N 0.987 *

## Data Availability

The data presented in this study are openly available in NCBI (GenBank accession numbers PX925626-29).

## Supplementary Materials

The following supporting information can be downloaded at: https://www.mdpi.com/article/10.3390/biology15070571/s1, Figure S1: Size comparison of mitochondrial genomic components across Hydrophiloidea. Bars represent the lengths (bp) of protein-coding genes (PCGs, concatenated), ribosomal RNA genes (*rrnL*, *rrnS*), control region (CR), and concatenated transfer RNA genes (tRNAs) for each species. Species are abbreviated as follows: Ag, *Amphiops globus*; Ba, *Berosus affinis*; Cb, *Cercyon borealis*; Cu1, *Cercyon unipunctatus* CJZSHRMP; Cu2, *Cercyon unipunctatus* CQMLYGP; Cu3, *Cercyon unipunctatus* CZDJDP; Cu4, *Cercyon unipunctatus* CZKXBSP; Cm, *Cryptopleurum minutum*; Cma, *Cymbiodyta marginella*; He, *Helochares* KT876891; Hel, *Helophorus* KX035139; Hr, *Helophorus rufipes*; Hf, *Hydrobius fuscipes*; Hc, *Hydrochus carinatus*; Hy, *Hydrochus* KT876892; Hy1, Hydrophilidae KT696213; Hy2,Hydrophilidae KT696219; Hy3, Hydrophilidae KT696220; Hy4, Hydrophilidae KT696222; Hy5, Hydrophilidae KT696224; Hy6, Hydrophilidae KT696262; Hb, *Hydrophilus bilineatus*; Sb, *Sphaeridium bipustulatum*; Sl, *Sphaeridium lunatum*; Sr, *Sternolophus rufipes*; Tr, *Tropisternus* NC_018349; Figure S2. Mean AT content of 13 protein-coding genes (PCGs) across six subfamilies of Hydrophiloidea. Bars represent mean AT content with standard deviation for Acidocerinae, Enochrinae, Helophorinae, Hydrochinae, Hydrophilinae, and Sphaeridiinae; Figure S3. Relative synonymous codon usage (RSCU) in mitochondrial protein-coding genes (PCGs). (A) Average RSCU across four newly sequenced *Cercyon unipunctatus* mitogenomes. (B) Average RSCU across all Hydrophiloidea mitogenomes. Codon families are color-coded by their corresponding amino acid. RSCU > 1.0 indicates preferential codon usage; Figure S4. Evolutionary rates and GC content of mitochondrial protein-coding genes (PCGs) in Hydrophiloidea. (A) Bar chart showing mean *Ka*/*Ks* ratio (left *Y*-axis) and corresponding mean GC content (right *Y*-axis, line plot) for each of the 13 PCGs. Error bars represent standard error. (B) Correlation analysis between *Ka*/*Ks* and GC content. Shaded area indicates 95% confidence interval. Each point represents one PCG; Figure S5. Comparative *Ka*/*Ks* ratio variation across Hydrophiloidea lineages. (A) *Ka*/*Ks* ratio variation among three families; (B) *Ka*/*Ks* ratio among four subfamilies of Hydrophiloidea. Error bars represent standard error; Figure S6. Relationship between effective number of codons (ENC) and GC3 content in Hydrophiloidea mitogenomes. The solid curve (ENC*) represents the expected ENC under pure mutational bias; Figure S7. Sequence alignment of the conserved *trnS2*-*nad1* intergenic spacer. Conserved bases across all 26 Hydrophiloidea species are highlighted. Newly sequenced species are shown in red. Color shading indicates subfamily-specific conservation: green (Hydrophilinae), cyan (Sphaeridiinae), brown (Helophorinae), pink (Hydrochinae); Figure S8. *Ka*/*Ks* ratio of PCGs among Hydrophiloidea species with different habitat types. Error bars represent standard error. Table S1. Collection details for newly sequenced *Cercyon unipunctatus* specimens; Table S2. Taxonomic classification and GenBank accession numbers of Hydrophiloidea species included in this study. Newly sequenced mitogenomes are indicated in bold; Table S3. Habitat type of Hydrophiloidea species included in this study. Newly sequenced mitogenomes are indicated in bold; Table S4. Substitution saturation tests (DAMBE) for phylogenetic datasets. Iss, index of substitution saturation; Iss.cS, critical Iss value; Table S5. The best partitioning schemes and substitution models selected by IQ-TREE for the three datasets; Table S6. Neutrality test results for seven protein-coding genes (PCGs) and five tRNA genes across three geographic populations of *Cercyon unipunctatus* (n = 10 per population); Table S7. Annotation of the four newly sequenced mitogenomes of *Cercyon unipunctatus*; Table S8. Codon usage statistics for 13 protein-coding genes (PCGs) across Hydrophiloidea mitogenomes. RSCU, relative synonymous codon usage; %, percentage of total count; AA, amino acid; Table S9. Maximal tandem repeat sequences in Hydrophiloidea mitochondrial control regions. Sequences correspond to colored numbered blocks marked with asterisks (*) in Figure 3. Newly sequenced species are indicated in bold; Table S10. Topology test results. DeltaL: logL difference from the maximal logl in the set; RELL: bootstrap proportion using RELL method; KH: one-sided Kishino–Hasegawa test; SH: Shimodaira–Hasegawa test; WKH: weighted KH test; WSH: weighted SH test; ELW: Expected Likelihood Weight; AU: approximately unbiased (AU) test.
