# Supplementary material for: Comparative Mitogenomic Analysis of Water Scavenger Beetles (Coleoptera: Hydrophiloidea) Provides Insights into Phylogeny and Adaptive Evolution
Source: Biology (Basel). 2026 Apr 2;15(7):571. doi: 10.3390/biology15070571 (PMC13072397; doi:10.3390/biology15070571)
Supplement: Supplementary file 1 [file biology-15-00571-s001.zip › Figure S7 Sequence alignment of the conserved trnS2-nad1.pdf]

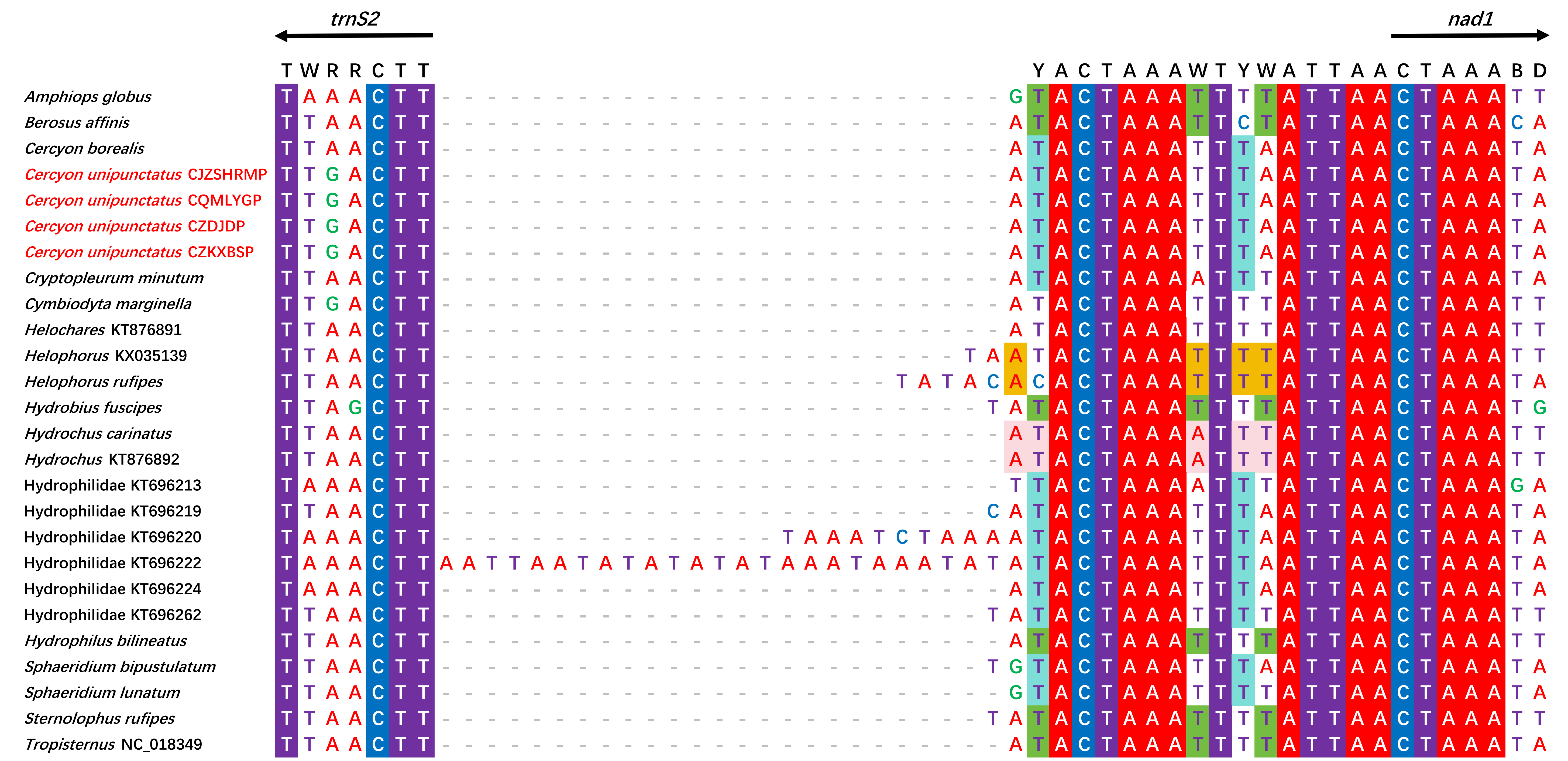
trnS2
nad1
T W R R C T T
Y A C T A A A W T Y W A T T A A C T A A A B D
Amphiops globus
Berosus affinis
Cercyon borealis
Cercyon unipunctatus CJZSHRMP
Cercyon unipunctatus CQMLYGP
Cercyon unipunctatus CZDJDP
Cercyon unipunctatus CZKXBSP
Cryptopleurum minutum
Cymbiodyta marginella
Helochares KT876891
Helophorus KX035139
Helophorus rufipes
Hydrobius fuscipes
Hydrochus carinatus
Hydrochus KT876892
Hydrophilidae KT696213
Hydrophilidae KT696219
Hydrophilidae KT696220
Hydrophilidae KT696222
Hydrophilidae KT696224
Hydrophilidae KT696262
Hydrophilus bilineatus
Sphaeridium bipustulatum
Sphaeridium lunatum
Sternolophus rufipes
Tropisternus NC_018349
