# Supplementary material for: Comparative Mitogenomic Analysis of Water Scavenger Beetles (Coleoptera: Hydrophiloidea) Provides Insights into Phylogeny and Adaptive Evolution
Source: Biology (Basel). 2026 Apr 2;15(7):571. doi: 10.3390/biology15070571 (PMC13072397; doi:10.3390/biology15070571)
Supplement: Supplementary file 1 [file biology-15-00571-s001.zip › Table S1 Collection details for newly sequenced.pdf]

Table S1 Collection details for newly sequenced *Cercyon unipunctatus* specimens.

Species	Sampling locality	Specimen voucher	Coordinate	Altitude (m)
Cercyon unipunctatus CQMLYGP	Qumalai County, Qinghai Province, China	CQMLYGP	34°12'N, 95°80'E	4140
Cercyon unipunctatus CJZSHRMP	Jiuzhi County, Qinghai Province, China	CJZSHRMP	33°44'N, 101°12'E	3938
Cercyon unipunctatus CZKXBSP	Zêkog County, Qinghai Province, China	CZKXBSP	35°10'N, 101°74'E	3674
Cercyon unipunctatus CZDJDP	Zadoi County, Qinghai Province, China	CZDJDP	32°78'N, 95°13'E	4453
