# Supplementary material for: Comparative Mitogenomic Analysis of Water Scavenger Beetles (Coleoptera: Hydrophiloidea) Provides Insights into Phylogeny and Adaptive Evolution
Source: Biology (Basel). 2026 Apr 2;15(7):571. doi: 10.3390/biology15070571 (PMC13072397; doi:10.3390/biology15070571)
Supplement: Supplementary file 1 [file biology-15-00571-s001.zip › Table S2 Information of Hydrophiloidea species used in this study.pdf]

Table S2 Taxonomic classification and GenBank accession numbers of Hydrophiloidea species included in this study. Newly sequenced mitogenomes are indicated in bold.

Famliy	Subfamliy	Species	Accession Number	Reference
Helophoridae	Helophorinae	*Helophorus* KX035139	KX035139	[25]
		Helophorus rufipes	MK692564	-
Hydrochidae	Hydrochinae	*Hydrochus carinatus*	NC_036270	-
		Hydrochus KT876892	KT876892	[25]
Hydrophilidae	Acidocerinae	*Helochares* KT876891	KT876891	[25]
	Enochrinae	*Cymbiodyta marginella*	KX087278	-
	Hydrophilinae	*Amphiops globus*	JX412726	-
		Berosus affinis	KT876883	[25]
		Hydrophilus bilineatus	LC875518	[8]
		Hydrobius fuscipes	MT862396	-
		Sternolophus rufipes	NC_072667	-
		Tropisternus NC_018349	NC_018349	[24]
	Sphaeridiinae	*Cercyon borealis*	KX087255	-
		***Cercyon unipunctatus* CQMLYGP**	PX925626	This study
		***Cercyon unipunctatus* CJZSHRMP**	PX925627	This study
		***Cercyon unipunctatus* CZKXBSP**	PX925628	This study
		***Cercyon unipunctatus* CZDJDP**	PX925629	This study
		Cryptopleurum minutum	KT780640	-
		Sphaeridium bipustulatum	NC_028612	-
		Sphaeridium lunatum	NC_063613	-
		Hydrophilidae KT696213	KT696213	-
		Hydrophilidae KT696219	KT696219	-
		Hydrophilidae KT696220	KT696220	-
		Hydrophilidae KT696222	KT696222	-
		Hydrophilidae KT696224	KT696224	-
		Hydrophilidae KT696262	KT696262	-
Histeridae	Saprininae	*Euspilotus scissus*	GU176344	[24]
	Histerinae	*Margarinotus merdarius*	NC_028603	-

References:

8. Juiki, N.; Daiki, O.; Sunahata, H.; Rikuto, O.; Sotaro, N.; Erina, F.; Naoya, T.; Kiyoshi, T.; Ohba, S.-y.; Takahashi, J.-i. First complete mitochondrial genome data of *Hydrophilus bilineatus* deciphered within the genus *Hydrophilus*. *Data in Brief* **2025**, *62*, doi:10.1016/j.dib.2025.111936.

24. Song, H.; Sheffield, N.C.; Cameron, S.L.; Miller, K.B.; Whiting, M.F. When phylogenetic assumptions are violated: base compositional heterogeneity and among-site rate variation in beetle mitochondrial phylogenomics. *Systematic Entomology* **2010**, *35*, 429-448, doi:10.1111/j.1365-3113.2009.00517.x.

25. Linard, B.; Arribas, P.; Andújar, C.; Crampton-Platt, A.; Vogler, A.P. Lessons from genome skimming of arthropod-preserving ethanol. *Molecular Ecology Resources* **2016**, *16*, 1365-1377, doi:10.1111/1755-0998.12539.
