# Supplementary material for: Comparative Mitogenomic Analysis of Water Scavenger Beetles (Coleoptera: Hydrophiloidea) Provides Insights into Phylogeny and Adaptive Evolution
Source: Biology (Basel). 2026 Apr 2;15(7):571. doi: 10.3390/biology15070571 (PMC13072397; doi:10.3390/biology15070571)
Supplement: Supplementary file 1 [file biology-15-00571-s001.zip › Table S3 Habitat type of Hydrophiloidea species included in this study.pdf]

Table S3 Habitat type of Hydrophiloidea species included in this study. Newly sequenced mitogenomes are indicated in bold.

Famliy	Subfamliy	Species	Habits	Reference
Helophoridae	Helophorinae	*Helophorus* KX035139	Adults are aquatic, while larvae are terrestrial.	[31]
		Helophorus rufipes	Adults are aquatic, while larvae are terrestrial	[31]
Hydrochidae	Hydrochinae	*Hydrochus carinatus*	Fully aquatic	[26]
		Hydrochus KT876892	Fully aquatic	[26]
Hydrophilidae	Acidocerinae	*Helochares* KT876891	Aquatic	[27]
	Enochrinae	*Cymbiodyta marginella*	Aquatic	[34]
	Hydrophilinae	*Amphiops globus*	Aquatic	[27]
		Berosus affinis	Fully aquatic	[30]
		Hydrophilus bilineatus	Aquatic	[27]
		Hydrobius fuscipes	Aquatic	[28]
		Sternolophus rufipes	Aquatic	[27]
		Tropisternus NC_018349	Aquatic	[33]
	Sphaeridiinae	*Cercyon borealis*	Terrestrial	[29]
		***Cercyon unipunctatus* CQMLYGP**	Terrestrial	[29]
		***Cercyon unipunctatus* CJZSHRMP**	Terrestrial	[29]
		***Cercyon unipunctatus* CZKXBSP**	Terrestrial	[29]
		***Cercyon unipunctatus* CZDJDP**	Terrestrial	[29]
		Cryptopleurum minutum	Terrestrial	[29]
		Sphaeridium bipustulatum	Terrestrial	[32]
		Sphaeridium lunatum	Terrestrial	[32]
		Hydrophilidae KT696213	Terrestrial	[27]
		Hydrophilidae KT696219	Terrestrial	[27]
		Hydrophilidae KT696220	Terrestrial	[27]
		Hydrophilidae KT696222	Terrestrial	[27]
		Hydrophilidae KT696224	Terrestrial	[27]
		Hydrophilidae KT696262	Terrestrial	[27]

References:

26. Alessandro, M. Coleoptera, Beetles. Volume 1: Morphology and Systematics. Archostemata, Adephaga, Myxophaga, Polyphaga partim. *Fragmenta Entomologica* **2017**, *49*, 192-192, doi:10.4081/fe.2017.265.
27. Fikáˇcek, M. Hydrophilidae Leach, 1815. In *Australian Beetles Volume 2: Archostemata, Myxophaga, Adephaga, Polyphaga (part)*, Ślipiński, A., Lawrence, J.F., Eds.; CSIRO Publishing: 2019; pp. 271-337.
28. Fossen, E.I.; Ekrem, T.; Nilsson, A.N.; Bergsten, J. Species delimitation in northern European water scavenger beetles of the genus *Hydrobius* (Coleoptera, Hydrophilidae). *Zookeys* **2016**, 71-120, doi:10.3897/zookeys.564.6558.
29. Jia, F.L.; Liang, Z.L.; Ryndevich, S.K.; Fikácek, M. Two new species and additional faunistic records of *Cercyon* Leach, 1817 from China (Coleoptera: Hydrophilidae). *Zootaxa* **2019**, *4565*, 501-514, doi:10.11646/zootaxa.4565.4.4.
30. Karaouzas, I.; Incekara, Ü. First record of the genus *Berosus* (Coleoptera: Hydrophilidae) in Crete island. *Entomologia hellenica* **2011**, *20*, 69-74, doi:10.12681/eh.11510.
31. Landin, J. Habitats, life histories, migration and dispersal by flight of two water-beetles *Helophorus brevipalpis* and *H. strigifrons* (Hydrophilidae). *Holarctic Ecology* **1980**, *3*, 190-201, doi:10.1111/j.1600-0587.1980.tb00725.x.
32. Proctor, M.C.F. A key to the British species of *Sphagnum. Transactions of the British Bryological Society* **1955**, *2*, 552-560, doi:10.1179/006813855804830001.

33. Spangler, P.J. A revision of the genus *Tropisternus* (Coleoptera: Hydrophilidae). Ph.D., University of Missouri - Columbia, United States -- Missouri, 1960.
34. Toussaint, E.F.A.; Short, A.E.Z. Historical biogeography of holarctic *Cymbiodyta* water scavenger beetles in the times of Cenozoic land bridge dispersal routes. *Insect Systematics and Diversity* **2019**, *3*, doi:10.1093/isd/ixz017.
