# Supplementary material for: Comparative Mitogenomic Analysis of Water Scavenger Beetles (Coleoptera: Hydrophiloidea) Provides Insights into Phylogeny and Adaptive Evolution
Source: Biology (Basel). 2026 Apr 2;15(7):571. doi: 10.3390/biology15070571 (PMC13072397; doi:10.3390/biology15070571)
Supplement: Supplementary file 1 [file biology-15-00571-s001.zip › Table S4 Substitution saturation tests (DAMBE) for phylogenetic datasets.pdf]

Table S4 Substitution saturation tests (DAMBE) for phylogenetic datasets. Iss, index of substitution saturation; Iss.cS, critical Iss value.

Dataset	Iss	Iss.cS	*P* value
atp6	0.2641	0.7360	＜0.0001
atp8	0.4600	0.5916	＜0.0001
cob	0.2760	0.7675	＜0.0001
cox1	0.2187	0.7802	＜0.0001
cox2	0.2769	0.7376	＜0.0001
cox3	0.2339	0.7468	＜0.0001
nad1	0.2886	0.7579	＜0.0001
nad2	0.4806	0.7612	＜0.0001
nad3	0.3658	0.6784	＜0.0001
nad4	0.3279	0.7748	＜0.0001
nad4L	0.3610	0.6588	＜0.0001
nad5	0.3045	0.7831	＜0.0001
nad6	0.5612	0.7103	＜0.0001
P123	0.3092	0.8381	＜0.0001
Position 1st	0.2470	0.8028	＜0.0001
Position 2nd	0.1263	0.8028	＜0.0001
Position 3rd	0.6372	0.8028	＜0.0001
P123R	0.3310	0.8427	＜0.0001
rrnS	0.4512	0.7488	＜0.0001
rrnL	0.4606	0.7753	＜0.0001
