# Supplementary material for: Comparative Mitogenomic Analysis of Water Scavenger Beetles (Coleoptera: Hydrophiloidea) Provides Insights into Phylogeny and Adaptive Evolution
Source: Biology (Basel). 2026 Apr 2;15(7):571. doi: 10.3390/biology15070571 (PMC13072397; doi:10.3390/biology15070571)
Supplement: Supplementary file 1 [file biology-15-00571-s001.zip › Table S5 The best partitioning schemes and substitution models selected.pdf]

Table S5 The best partitioning schemes and substitution models selected by IQ-TREE for the three datasets.

Dataset	Subset	Best-fit scheme	Model
P123	P1	*atp6*_pos1, *cob*_pos1	GTR+F+I+G4
	P2	*atp6*_pos2, *cob*_pos2, *cox1*_pos2, *cox2*_pos2, *cox3*_pos2	GTR+F+I+G4
	P3	*atp6*_pos3, *nad3*_pos3	HKY+F+I+G4
	P4	*atp8*_pos1, *nad2*_pos1, *nad3*_pos1	TIM2+F+I+R3
	P5	*atp8*_pos2, *nad2*_pos2, *nad3*_pos2, *nad6*_pos2	TVM+F+I+G4
	P6	*atp8*_pos3, *nad2*_pos3, *nad6*_pos3	HKY+F+I+R3
	P7	*cob*_pos3, *cox1*_pos3, *cox2*_pos3, *cox3*_pos3	K3Pu+F+I+R3
	P8	*cox1*_pos1, *cox2*_pos1, *cox3*_pos1	GTR+F+I+G4
	P9	*nad1*_pos1, *nad4*_pos1, *nad4L*_pos1, *nad5*_pos1	TIM2+F+I+G4
	P10	*nad1*_pos2, *nad4*_pos2, *nad4L*_pos2, *nad5*_pos2	GTR+F+I+R2
	P11	*nad1*_pos3, *nad4*_pos3, *nad4L*_pos3, *nad5*_pos3	K3Pu+F+I+R3
	P12	*nad6*_pos1	TIM2+F+I+G4
P123R	P1	*atp6*_pos1, *atp8*_pos1, *cob*_pos1, *nad3*_pos1	GTR+F+I+G4
	P2	*atp6*_pos2, *cob*_pos2, *cox1*_pos2, *cox2*_pos2, *cox3*_pos2, *nad1*_pos2	TVM+F+I+G4
	P3	*atp6*_pos3, *nad3*_pos3	HKY+F+I+G4
	P4	*atp8*_pos2, *nad2*_pos2, *nad6*_pos2	TVM+F+I+G4
	P5	*atp8*_pos3	HKY+F+R2
	P6	*cob*_pos3, *cox1*_pos3, *cox2*_pos3, *cox3*_pos3	TIM2+F+I+R3
	P7	*cox1*_pos1, *cox2*_pos1, *cox3*_pos1	GTR+F+I+G4
	P8	*nad1*_pos1, *nad4*_pos1, *nad4L*_pos1, *nad5*_pos1	TIM2+F+I+G4
	P9	*nad1*_pos3, *nad4*_pos3, *nad4L*_pos3, *nad5*_pos3	HKY+F+I+G4

	P10	*nad2*_pos1	TIM2+F+I+R3
	P11	*nad2*_pos3, *nad6*_pos3	HKY+F+I+R3
	P12	*nad3*_pos2, *nad4*_pos2, *nad4L*_pos2, *nad5*_pos2	GTR+F+I+R2
	P13	*nad6*_pos1	TIM2+F+I+G4
	P14	*rrnL*, *rrnS*	GTR+F+I+G4
P123AA	P1	*atp6*, *cob*, *cox1*, *cox2*, *cox3*	MTART+I+R3
	P2	*atp8*, *nad1*, *nad3*, *nad4*, *nad4L*, *nad5*	MTINV+R4
	P3	*nad2*, *nad6*	MTZOA+F+I+G4
