# Supplementary material for: Comparative Mitogenomic Analysis of Water Scavenger Beetles (Coleoptera: Hydrophiloidea) Provides Insights into Phylogeny and Adaptive Evolution
Source: Biology (Basel). 2026 Apr 2;15(7):571. doi: 10.3390/biology15070571 (PMC13072397; doi:10.3390/biology15070571)
Supplement: Supplementary file 1 [file biology-15-00571-s001.zip › Table S6 Neutrality test results.pdf]

Table S6 Neutrality test results for seven protein-coding genes (PCGs) and five tRNA genes across three geographic populations of *Cercyon unipunctatus* (n = 10 per population).

Species	Genes	*S*	*Pi*	*Hd*	Tajima' s *D*	*P*	Fu' s Fs	Fu & Li' s *D*	*P*	Fu & Li' s *F*	*P*
CJZSHRMP	*atp6*	1	0.00051	0.200	-1.11173	*P*>0.10	-0.339	-1.24341	*P*>0.10	-1.34668	*P*>0.10
CQMLYGP		1	0.00100	0.389	0.15647	*P*>0.10	0.477	0.84040	*P*>0.10	0.74837	*P*>0.10
CZDJDP		2	0.00093	0.345	-1.42961	*P*>0.10	-1.246	-1.65766	*P*>0.10	-1.79737	*P*>0.10
All		3	0.00082	0.303	-1.36029	*P*>0.10	-2.243	-1.47512	*P*>0.10	-1.66994	*P*>0.10
CJZSHRMP	*atp8*	1	0.00131	0.2	-1.11173	*P*>0.10	-0.339	-1.24341	*P*>0.10	-1.34668	*P*>0.10
CQMLYGP		0	0	0	-	-	-	-	-	-	-
CZDJDP		1	0.00119	0.182	-1.12850	*P*>0.10	-0.410	-1.28946	*P*>0.10	-1.39919	*P*>0.10
All		2	0.00087	0.131	-1.50738	*P*>0.10	-2.355	-2.28108	0.10>*P*>0.05	-2.21460	0.10>*P*>0.05
CJZSHRMP	*cox1*	180	0.07349	0.722	-1.71134	0.10>*P*>0.05	8.692	-89921	0.10>*P*>0.05	-2.05026	0.10>*P*>0.05
CQMLYGP		104	0.0429	0.75	-1.86272	*P*<0.01	7.856	-2.07793	*P*<0.02	-2.23434	*P*<0.02
CZDJDP		202	0.11533	0.857	-1.31345	*P*>0.10	6.467	-1.02877	*P*>0.10	-1.36763	*P*>0.10
All		249	0.07075	0.77	-1.81111	*P*<0.05	10.262	-1.05464	*P*>0.10	-1.52859	*P*>0.10
CJZSHRMP	*cox2*	2	0.00058	0.378	-1.40085	*P*>0.10	-1.164	-1.58662	*P*>0.10	-1.71902	*P*>0.10
CQMLYGP		97	0.0285	0.378	-2.1539	*P*<0.001	11.932	-2.55758	*P*<0.02	-2.777	*P*<0.02
CZDJDP		3	0.00122	0.691	-0.62785	*P*>0.10	-1.116	0.12672	*P*>0.10	-0.0677	*P*>0.10
All		101	0.00923	0.47	-2.81589	*P*<0.001	4.612	-5.57040	*P*<0.02	-5.15401	*P*<0.02
CJZSHRMP	*cox3*	0	0	0	-	-	-	-	-	-	-
CQMLYGP		2	0.00119	0.733	1.03299	*P*>0.10	0.345	1.02623	*P*>0.10	1.14601	*P*>0.10
CZDJDP		3	0.00204	0.533	1.83053	0.10>*P*>0.05	3.338	1.15417	*P*>0.10	1.47337	0.10>*P*>0.05
All		4	0.00140	0.687	0.22676	*P*>0.10	0.643	1.05802	*P*>0.10	0.94642	*P*>0.10
CJZSHRMP	*nad3*	0	0	0	-	-	-	-	-	-	-
CQMLYGP		3	0.00326	0.714	-0.30187	*P*>0.10	0.263	-0.519	*P*>0.10	-0.50749	*P*>0.10
CZDJDP		2	0.00285	0.5	-0.7099	*P*>0.10	1.099	-0.70990	*P*>0.10	-0.60427	*P*>0.10
All		3	0.00223	0.523	-0.2794	*P*>0.10	0.588	1.0236	*P*>0.10	0.77112	*P*>0.10
CJZSHRMP	*nad5*	22	0.00684	0.533	2.40538	*P*<0.01	12.445	1.56704	*P*<0.02	2.00243	*P*<0.02
CQMLYGP		3	0.00082	0.733	1.15198	*P*>0.10	1.160	1.15417	*P*>0.10	1.28920	*P*>0.10
CZDJDP		31	0.00489	0.378	-1.13225	*P*>0.10	7.159	-0.63328	*P*>0.10	-0.85515	*P*>0.10
All		48	0.00604	0.768	-0.54104	*P*>0.10	9.532	-0.64872	*P*>0.10	-0.72404	*P*>0.10
CJZSHRMP	*trnA*	0	0	0	-	-	-	-	-	-	-
CQMLYGP		0	0	0	-	-	-	-	-	-	-

CZDJDP		0	0	0	-	-	-	-	-	-	-
All		0	0	0	-	-	-	-	-	-	-
CJZSHRMP	*trnC*	0	0	0	-	-	-	-	-	-	-
CQMLYGP		0	0	0	-	-	-	-	-	-	-
CZDJDP		1	0.00298	0.182	-1.1285	*P*>0.10	-0.41	-1.28946	*P*>0.10	-1.39919	*P*>0.10
All		1	0.00102	0.063	-1.14244	*P*>0.10	-1.265	-1.70335	*P*>0.10	-1.66118	*P*>0.10
CJZSHRMP	*trnG*	0	0	0	-	-	-	-	-	-	-
CQMLYGP		2	0.00606	0.2	-1.40085	*P*>0.10	0.586	-1.58662	*P*>0.10	-1.71902	*P*>0.10
CZDJDP		0	0	0	-	-	-	-	-	-	-
All		2	0.00184	0.061	-1.5017	*P*>0.10	-0.482	-2.33081	0.10>*P*>0.05	-2.25846	0.10>*P*>0.05
CJZSHRMP	*trnR*	0	0	0	-	-	-	-	-	-	-
CQMLYGP		0	0	0	-	-	-	-	-	-	-
CZDJDP		0	0	0	-	-	-	-	-	-	-
All		0	0	0	-	-	-	-	-	-	-
CJZSHRMP	*trnW*	0	0	0	-	-	-	-	-	-	-
CQMLYGP		3	0.00882	0.2	-1.56222	0.10>*P*>0.05	1.225	-1.78443	*P*>0.10	-1.9338	*P*>0.10
CZDJDP		0	0	0	-	-	-	-	-	-	-
All		3	0.00267	0.061	-1.7282	0.10>*P*>0.05	0.04	-2.75371	*P*<0.05	-2.84733	*P*<0.05
