# Supplementary material for: Comparative Mitogenomic Analysis of Water Scavenger Beetles (Coleoptera: Hydrophiloidea) Provides Insights into Phylogeny and Adaptive Evolution
Source: Biology (Basel). 2026 Apr 2;15(7):571. doi: 10.3390/biology15070571 (PMC13072397; doi:10.3390/biology15070571)
Supplement: Supplementary file 1 [file biology-15-00571-s001.zip › Table S7 Annotation of the four newly sequenced mitogenomes.pdf]

Table S7A Annotation of the newly sequenced mitogenomes of *Cercyon unipunctatus* CJZSHRMP.

Name	Strand	Position		Length(bp)	Intergenic nucleotides(bp)	Codon		
						Anti	Start	Stop
trnM	+	1	69	69	0	CAT		
nad2	+	70	1072	1003	0		ATT	T
trnW	+	1073	1140	68	0	TCA		
trnC	-	1133	1193	61	-8	GCA		
trnY	-	1195	1260	66	1	GTA		
cox1	+	1253	2792	1540	-8		ATC	T
trnL2	+	2793	2856	64	0	TAA		
cox2	+	2857	3544	688	0		ATT	T
trnK	+	3545	3615	71	0	CTT		
trnD	+	3615	3681	67	-1	GTC		
atp8	+	3682	3837	156	0		ATT	TAA
atp6	+	3831	4504	674	-7		ATG	TA
cox3	+	4505	5291	787	0		ATG	T
trnG	+	5292	5357	66	0	TCC		
nad3	+	5361	5709	349	3		ATC	T
trnA	+	5710	5774	65	0	TGC		
trnR	+	5774	5839	66	-1	TCG		
trnN	+	5840	5904	65	0	GTT		
trnS1	+	5905	5971	67	0	TCT		
trnE	+	5973	6040	68	1	TTC		
trnF	-	6039	6105	67	-2	GAA		
nad5	-	6106	7823	1718	0		ATG	TA
trnH	-	7825	7889	65	1	GTG		
nad4	-	7890	9225	1336	0		ATG	T
nad4L	-	9219	9503	285	-7		ATG	TAA
trnT	+	9506	9570	65	2	TGT		
trnP	-	9570	9634	65	-1	TGG		
nad6	+	9636	10120	485	1		ATC	TA
cob	+	10121	11261	1141	0		ATG	T
trnS2	+	11262	11328	67	0	TGA		
nad1	-	11346	12296	951	17		TTG	TAG
trnL1	-	12298	12360	63	1	TAG		
l-rRNA	-	12361	13641	1281	0			
trnV	-	13642	13711	70	0	TAC		
s-rRNA	-	13712	14490	779	0			
trnI	+	16678	16742	65	0	GAT		
trnQ	-	16740	16807	68	-3	TTG		

Table S7B Annotation of the newly sequenced mitogenomes of *Cercyon unipunctatus* CQMLYGP.

Name	Strand	Position		Length(bp)	Intergenic nucleotides(bp)	Codon		
						Anti	Start	Stop
trnI	+	1	65	65	0	GAT		
trnQ	-	63	131	69	-3	TTG		
trnM	+	131	199	69	-1	CAT		
nad2	+	200	1202	1003	0		ATT	T
trnW	+	1203	1270	68	0	TCA		
trnC	-	1263	1323	61	-8	GCA		
trnY	-	1325	1390	66	1	GTA		
cox1	+	1383	2922	1540	-8		ATC	T
trnL2	+	2923	2986	64	0	TAA		
cox2	+	2987	3674	688	0		ATT	T
trnK	+	3675	3745	71	0	CTT		
trnD	+	3745	3811	67	-1	GTC		
atp8	+	3812	3967	156	0		ATT	TAA
atp6	+	3961	4634	674	-7		ATG	TA
cox3	+	4635	5421	787	0		ATG	T
trnG	+	5422	5487	66	0	TCC		
nad3	+	5491	5839	349	3		ATC	T
trnA	+	5840	5904	65	0	TGC		
trnR	+	5904	5969	66	-1	TCG		
trnN	+	5970	6034	65	0	GTT		
trnS1	+	6035	6101	67	0	TCT		
trnE	+	6103	6170	68	1	TTC		
trnF	-	6169	6235	67	-2	GAA		
nad5	-	6236	7953	1718	0		ATG	TA
trnH	-	7955	8019	65	1	GTG		
nad4	-	8020	9355	1336	0		ATG	T
nad4L	-	9349	9633	285	-7		ATG	TAA
trnT	+	9636	9700	65	2	TGT		
trnP	-	9700	9764	65	-1	TGG		
nad6	+	9766	10250	485	1		ATC	TA
cob	+	10251	11391	1141	0		ATG	T
trnS2	+	11392	11458	67	0	TGA		
nad1	-	11476	12426	951	17		TTG	TAG
trnL1	-	12428	12490	63	1	TAG		
l-rRNA	-	12491	13771	1281	0			
trnV	-	13772	13841	70	0	TAC		
s-rRNA	-	13842	14620	779	0			

Table S7C Annotation of the newly sequenced mitogenomes of *Cercyon unipunctatus* CZDJDP.

Name	Strand	Position		Length(bp)	Intergenic nucleotides(bp)	Codon		
						Anti	Start	Stop
trnI	+	1	65	65	0	GAT		
trnQ	-	63	131	69	-3	TTG		
trnM	+	131	199	69	-1	CAT		
nad2	+	200	1202	1003	0		ATT	T
trnW	+	1203	1270	68	0	TCA		
trnC	-	1263	1323	61	-8	GCA		
trnY	-	1325	1390	66	1	GTA		
cox1	+	1383	2922	1540	-8		ATC	T
trnL2	+	2923	2986	64	0	TAA		
cox2	+	2987	3674	688	0		ATT	T
trnK	+	3675	3745	71	0	CTT		
trnD	+	3745	3811	67	-1	GTC		
atp8	+	3812	3967	156	0		ATT	TAA
atp6	+	3961	4634	674	-7		ATG	TA
cox3	+	4635	5421	787	0		ATG	T
trnG	+	5422	5487	66	0	TCC		
nad3	+	5491	5839	349	3		ATC	T
trnA	+	5840	5904	65	0	TGC		
trnR	+	5904	5969	66	-1	TCG		
trnN	+	5970	6034	65	0	GTT		
trnS1	+	6035	6101	67	0	TCT		
trnE	+	6103	6170	68	1	TTC		
trnF	-	6169	6235	67	-2	GAA		
nad5	-	6236	7953	1718	0		ATG	TA
trnH	-	7955	8019	65	1	GTG		
nad4	-	8020	9355	1336	0		ATG	T
nad4L	-	9349	9633	285	-7		ATG	TAA
trnT	+	9636	9700	65	2	TGT		
trnP	-	9700	9764	65	-1	TGG		
nad6	+	9766	10250	485	1		ATC	TA
cob	+	10251	11391	1141	0		ATG	T
trnS2	+	11392	11458	67	0	TGA		
nad1	-	11476	12426	951	17		TTG	TAG
trnL1	-	12428	12490	63	1	TAG		
l-rRNA	-	12491	13771	1281	0			
trnV	-	13772	13841	70	0	TAC		
s-rRNA	-	13842	14620	779	0			

Table S7D Annotation of the newly sequenced mitogenomes of *Cercyon unipunctatus* CZKXBSP.

Name	Strand	Position		Length(bp)	Intergenic nucleotides(bp)	Codon		
						Anti	Start	Stop
trnI	+	1	65	65	0	GAT		
trnQ	-	63	131	69	-3	TTG		
trnM	+	131	199	69	-1	CAT		
nad2	+	200	1202	1003	0		ATT	T
trnW	+	1203	1270	68	0	TCA		
trnC	-	1263	1323	61	-8	GCA		
trnY	-	1325	1390	66	1	GTA		
cox1	+	1383	2922	1540	-8		ATC	T
trnL2	+	2923	2986	64	0	TAA		
cox2	+	2987	3674	688	0		ATT	T
trnK	+	3675	3745	71	0	CTT		
trnD	+	3745	3811	67	-1	GTC		
atp8	+	3812	3967	156	0		ATT	TAA
atp6	+	3961	4634	674	-7		ATG	TA
cox3	+	4635	5421	787	0		ATG	T
trnG	+	5422	5487	66	0	TCC		
nad3	+	5491	5839	349	3		ATC	T
trnA	+	5840	5904	65	0	TGC		
trnR	+	5904	5969	66	-1	TCG		
trnN	+	5970	6034	65	0	GTT		
trnS1	+	6035	6101	67	0	TCT		
trnE	+	6103	6170	68	1	TTC		
trnF	-	6169	6235	67	-2	GAA		
nad5	-	6236	7953	1718	0		ATG	TA
trnH	-	7955	8019	65	1	GTG		
nad4	-	8020	9355	1336	0		ATG	T
nad4L	-	9349	9633	285	-7		ATG	TAA
trnT	+	9636	9700	65	2	TGT		
trnP	-	9700	9764	65	-1	TGG		
nad6	+	9766	10250	485	1		ATC	TA
cob	+	10251	11391	1141	0		ATG	T
trnS2	+	11392	11458	67	0	TGA		
nad1	-	11476	12426	951	17		TTG	TAG
trnL1	-	12428	12490	63	1	TAG		
l-rRNA	-	12491	13771	1281	0			
trnV	-	13772	13841	70	0	TAC		
s-rRNA	-	13842	14620	779	0			
