# Supplementary material for: Comparative Mitogenomic Analysis of Water Scavenger Beetles (Coleoptera: Hydrophiloidea) Provides Insights into Phylogeny and Adaptive Evolution
Source: Biology (Basel). 2026 Apr 2;15(7):571. doi: 10.3390/biology15070571 (PMC13072397; doi:10.3390/biology15070571)
Supplement: Supplementary file 1 [file biology-15-00571-s001.zip › Table S9 Maximal tandem repeat sequences.pdf]

Table S9 Hydrophiloidea mitochondrial genome control region maximal tandem repeat sequence, corresponding to the colored numbered blocks marked with * in Figure 3. Newly sequenced species are indicated in bold.

Species	Repeat sequence	Length	Perfect repetition
Amphiops globus	ATCAAAGCAAGAAAGACG	18	2
Berosus affinis	AATAATCCCAAGAAACCTGACGGATTACTATAAATATAGAAAACCAAATTGCTACAGATAAAAC<br>TGTAATTCTCCAGTAAACCCTTATAGCTTAAGTATCAAAAATAAAAGCTTTAGATATTGAAAGTA<br>TTTAAATAGACAAAACTGCCCCTAATAAAGTCTAATAAATA	170	2
Cercyon borealis	AATAAAACTATCTCTGGATCAAAATTAAACTGCTATCTCCCACTTATAGCAATTTGAAAACTA	63	2
***Cercyon unipunctatus* CJZSHRMP**	ATAAAATCAAAATTAAACCACAACTACCTCAGTAAGATACAAATAAAACTATCTCT	56	2
***Cercyon unipunctatus* CQMLYGP**	TCGCTATCTCCCACTTATAGCAATTTGAAAACTAGATAAAATCAAAATTAAACCACAACTACCTC<br>AGTAAGATACAAATAAAACTATCTCT	91	2
***Cercyon unipunctatus* CZDJDP**	AAAACTAAATAAAATCAAAATTAAACCACAACTACCTCAGTAAGATACAAATAAAACTATCTCT	64	2
***Cercyon unipunctatus* CZKXBSP**	CGCTATCTCCCACTTATAGCAATTTGAAAACTAGATAAAATCAAAATTAAACCACAACTACCTC<br>AGTAAGATACAAATAAAACTATCTCTA	91	2
Cryptopleurum minutum	CCAAACTATACTCATAGCCTATGAAACCAAACAAAATCAAAATAAACCATATATTGAACATAAA<br>TTTACAAATAGCAAAGCTATCTCCCCACGTAAACTCATGA	104	3
Cymbiodyta marginella	CCCAGCTATCTGGCCTACCTAGCAACCTAGCCTAGTCAAGTTAACCTG	48	2
Helochares KT876891	CCTTTCCCTCAGGTCTCGCCTCGATCGCAAGCTCCGCCCAGCTCGCGCGCCTTTCCCTCAGGTCT<br>CGCCTCGATCGCAAGCTCCGCCCAGCTCGCGCGCCTTTCCCTCAGGTCTCGCCTC	120	2
Helophorus KX035139	TAAACATAAAAT	12	2
Helophorus rufipes	ATTTTAATTTTAGAATTGCCCACATCTAAAATTAAACTTTGAAAAACCAAATAAACCAAATTTTA<br>AATTTTAATTTAGAATTGCCCACA	89	2
Hydrobius fuscipes	ATCTGGAATAT	11	2
Hydrochus carinatus	TCTTTTTTTTTTTTTT	16	2
Hydrochus KT876892	AAATTTAACTAACCCTAAATTAAAAAACTATCCACTCTACAAACTCTAATATTTCAACCTCGAAT<br>TATTTAAATTACCCCCAAATTTAACTAATCCTAAATTAAAAAACTATCCACTCTACAAACTCTAA<br>TCTTCAACCTCGAATTATTTAAATTACCCCCAAATTTAACTAACCCTAAATTAAAAAA	188	2
Hydrophilidae KT696213	ACTTTTATATTAA	13	2
Hydrophilidae KT696219	GCTATCTGCCCTAGTAAAATCCACATAATTCAGTAAAATTACATGATACTAAAACTAATTATTAA<br>TTTCATCAGCTATCTGCCCTAGTAAAATCCACATAATTCAGTAAAATTACATGATACTAAAACTA	146	2

	ATTATTAATTTCATCA		
Hydrophilidae KT696220	CCTAACAATATTTCATTTTACTCAAATAATTAATCCTAAACAACATAAAACAAATGTAACCCCCA<br>ATTAAAAATATACCAAAATTAATACTACCCCTAAATTAATTTTGATAAAATTTTTAA	122	2
Hydrophilidae KT696222	TATAAGATTAATAAGCAATCTTCCCCTTACCCAATAAAAAATATAAGTAAATAAAAAATTAAAG<br>CTTGAACTCAACATAAAATAAAAAAAACATCTTTAAATAACCTTTATATCCCCTATAAAAATAA<br>AAATTTTATTTTTATTATAAAACTATTTATAAGATTAATAAGCAATCTTCCCCTTACCCAATAAA<br>AAATATAAGTAAATAAAAAATTAAAGCTTGAACTCAACATAAAATAAAAAAAA	246	2
Hydrophilidae KT696224	AAATAACCCTACAATCCACTAATAAACAAAATATCAAATTATAAACTAATCAACCCCTAGACTA<br>ATTTTCCAACCTAAATATATAAAAAAACAAATACCAAATCACATTTTGTCTCCTAAATCAAACTA<br>AAATAAATAAAACCTCTAATTAAATCATCTCATTTAAACTAATTTACCCCTAAATTAATTT	190	2
Hydrophilidae KT696262	CTTAATCTTCCCAAATTAAA	20	2
Hydrophilus bilineatus	CCCTCCCAAGCTAATTGTGCTAACGCCGACGAACGTTGCCTCGACGAGCCCTCCCAAGCTCACC<br>GCGCTGACGCCGACGACCGT	84	2
Sphaeridium bipustulatum	TTTTTTTTTTTTTTCAA	17	2
Sphaeridium lunatum	TAGAATTCCTAAC	13	2
Sternolophus rufipes	ATTCAACTCCCATAAAACCCAAAACTCAAGCATTGTTTCAAAACTAGCTGTGCTGTAG	58	2
Tropisternus NC_018349	TAGACCTCCCAATC	14	2
