# Supplementary material for: Comparative Mitogenomic Analysis of Water Scavenger Beetles (Coleoptera: Hydrophiloidea) Provides Insights into Phylogeny and Adaptive Evolution
Source: Biology (Basel). 2026 Apr 2;15(7):571. doi: 10.3390/biology15070571 (PMC13072397; doi:10.3390/biology15070571)
Supplement: Supplementary file 1 [file biology-15-00571-s001.zip › Table S10 Topology test results.pdf]

Table S10 Topology test results. DeltaL: logL difference from the maximal logl in the set; RELL : bootstrap proportion using RELL method; KH: one sided Kishino-Hasegawa test; SH: Shimodaira-Hasegawa test; WKH: weighted KH test; WSH: weighted SH test; ELW: Expected Likelihood Weight; AU:approximately unbiased (AU) test.

Dataset	Topology	Phylogeny	logL	deltaL	KH	SH	WKH	WSH	ELW	AU
P123	((Helophoridae, Hydrochidae), Hydrophilidae);	P1	-145136.3188	0	0.95	1	0.95	0.95	0.957	0.955
	(Hydrochidae, (Helophoridae, Hydrophilidae));	P2	-145165.0765	28.758	0.05	0.05	0.05	0.05	0.0434	0.0447
P123AA	((Helophoridae, Hydrochidae), Hydrophilidae);	P1	-62378.12866	0	0.524	1	0.524	0.524	0.515	0.554
	(Hydrochidae, (Helophoridae, Hydrophilidae));	P2	-62378.52916	0.4005	0.476	0.476	0.476	0.476	0.485	0.446
P123R	((Helophoridae, Hydrochidae), Hydrophilidae);	P1	-170777.8483	0	0.964	1	0.964	0.964	0.969	0.961
	(Hydrochidae, (Helophoridae, Hydrophilidae));	P2	-170812.3439	34.496	0.036	0.036	0.036	0.036	0.0313	0.0392
