# Supplementary figures and images for: Comparative Mitogenomic Analysis of Water Scavenger Beetles (Coleoptera: Hydrophiloidea) Provides Insights into Phylogeny and Adaptive Evolution
Source: Biology (Basel). 2026 Apr 2;15(7):571. doi: 10.3390/biology15070571 (PMC13072397; doi:10.3390/biology15070571)

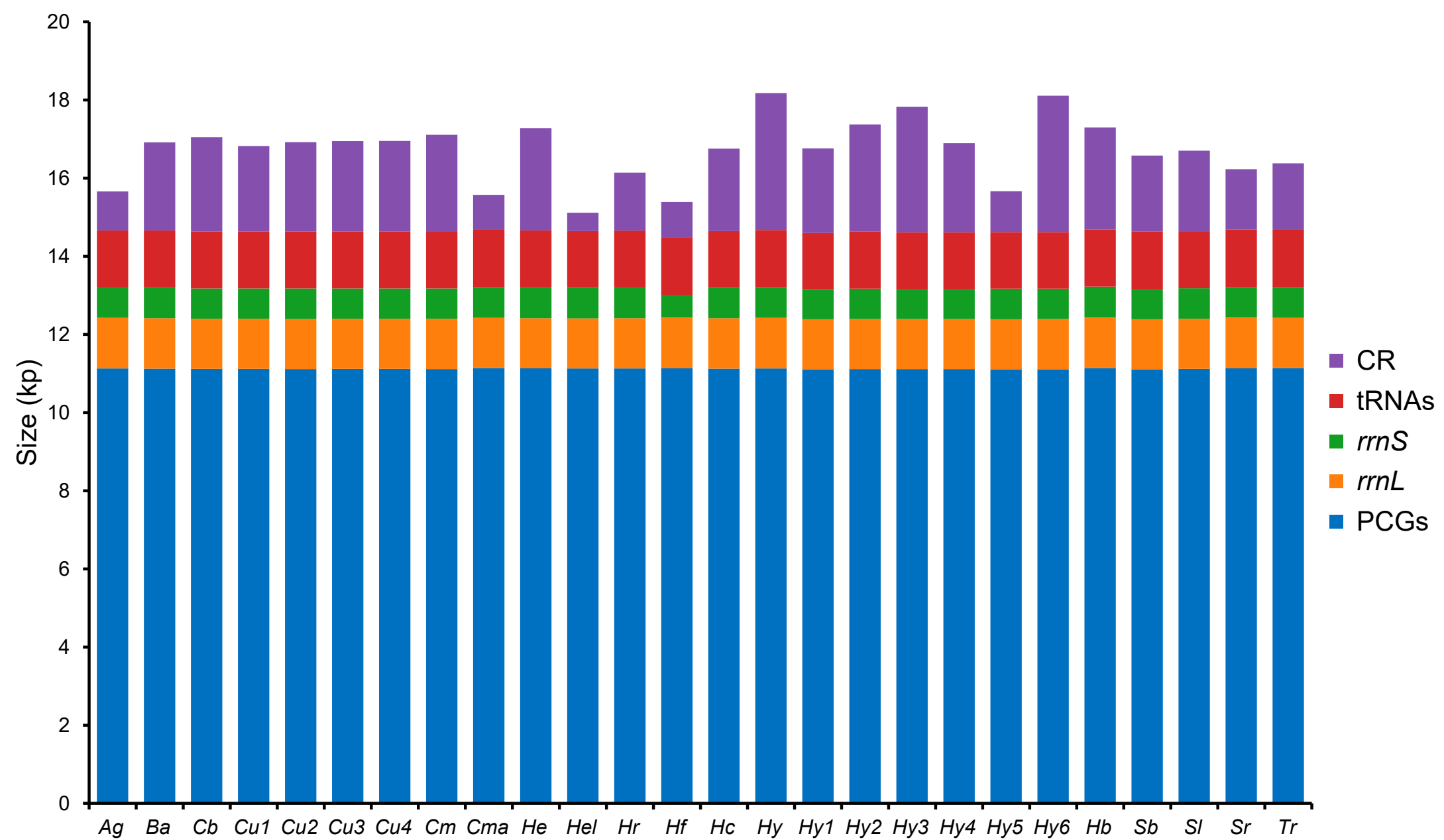

Supplement: Supplementary file 1 [file biology-15-00571-s001.zip › Figure S1 Size comparison of mitochondrial genomic.pdf]

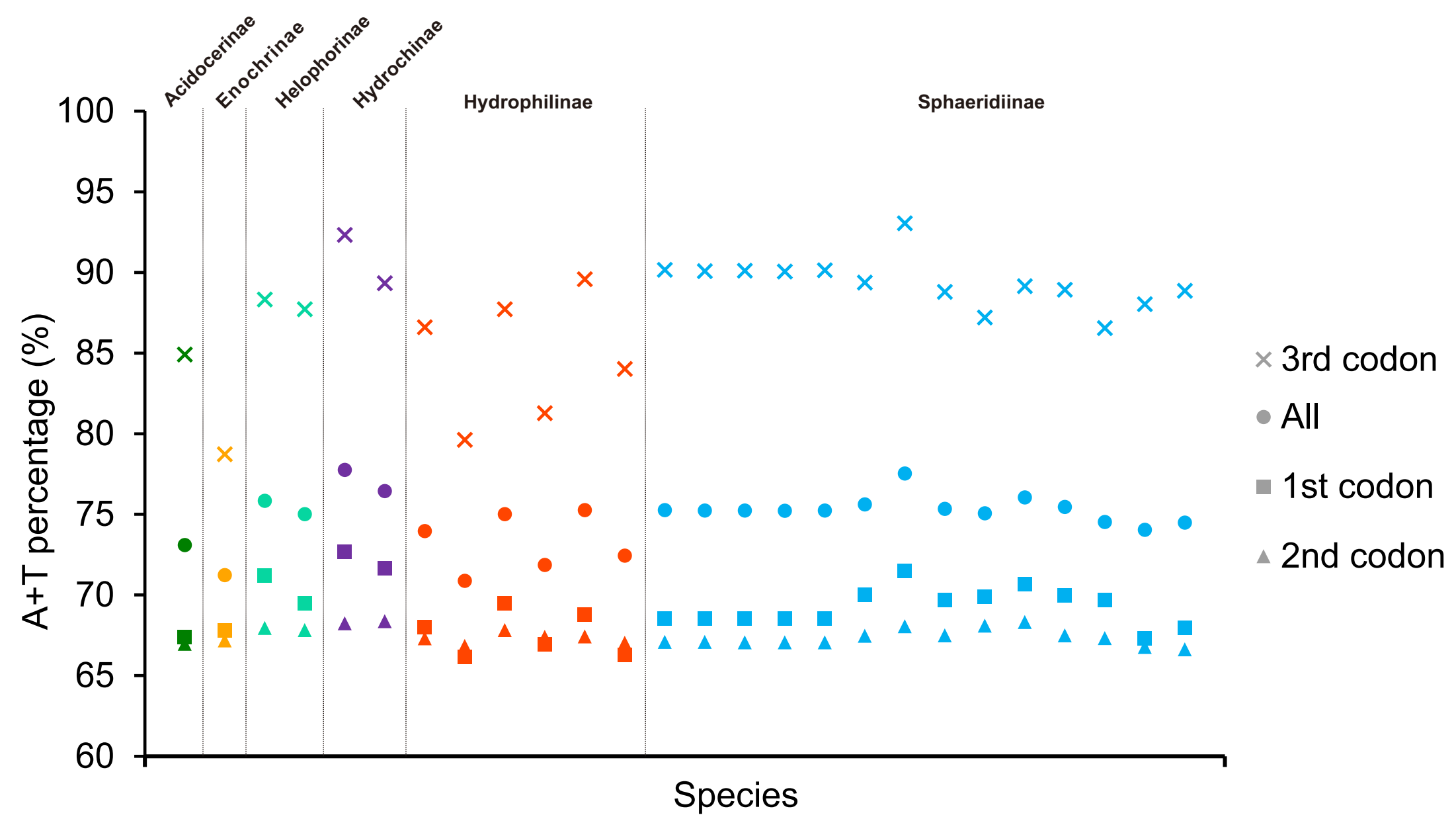

Supplement: Supplementary file 1 [file biology-15-00571-s001.zip › Figure S2 Mean A+T content of 13 protein-coding genes (PCGs).pdf]

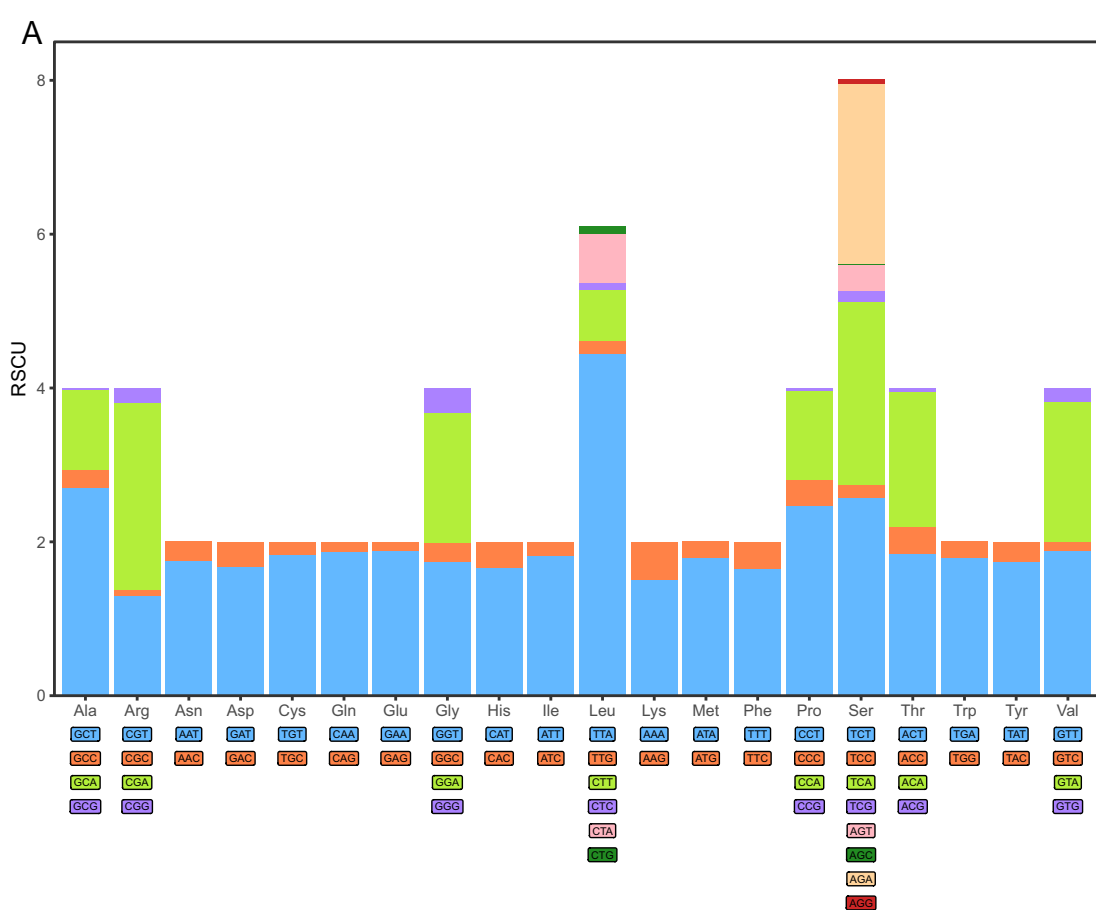

***Cercyon unipunctatus***

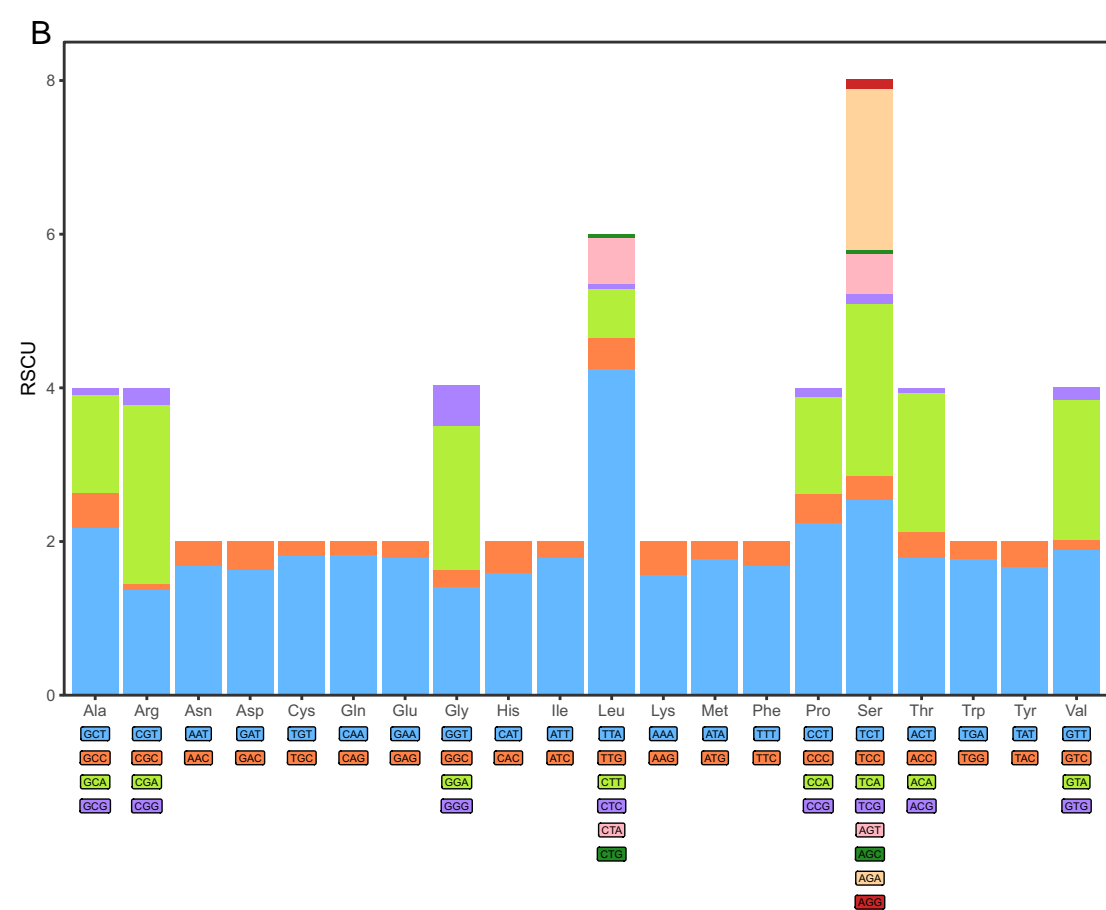

## Hydrophiloidea

Supplement: Supplementary file 1 [file biology-15-00571-s001.zip › Figure S3 Relative Synonymous Codon Usage (RSCU).pdf]

A

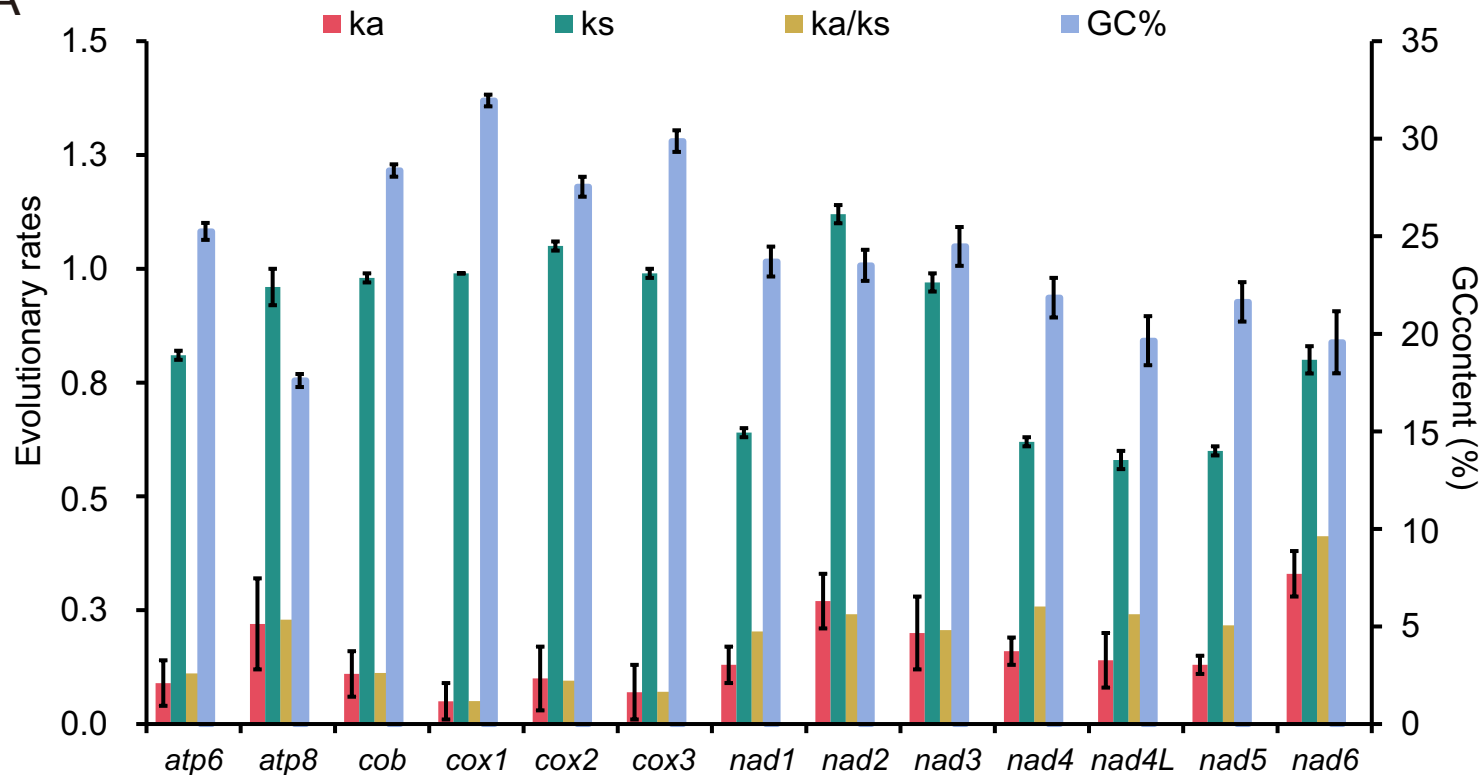

B

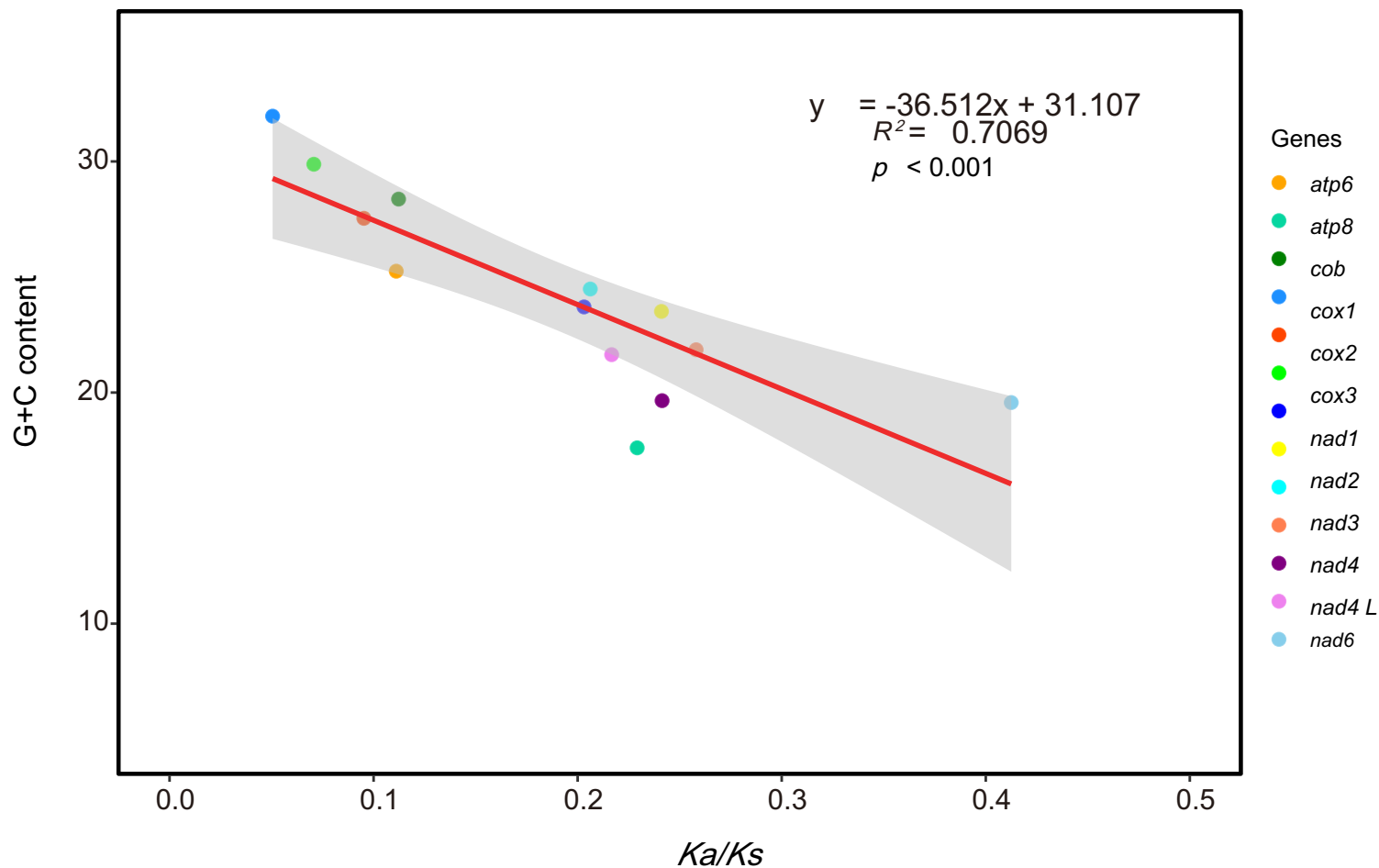

Supplement: Supplementary file 1 [file biology-15-00571-s001.zip › Figure S4 Evolutionary rates and GC content of mitochondrial PCGs.pdf]

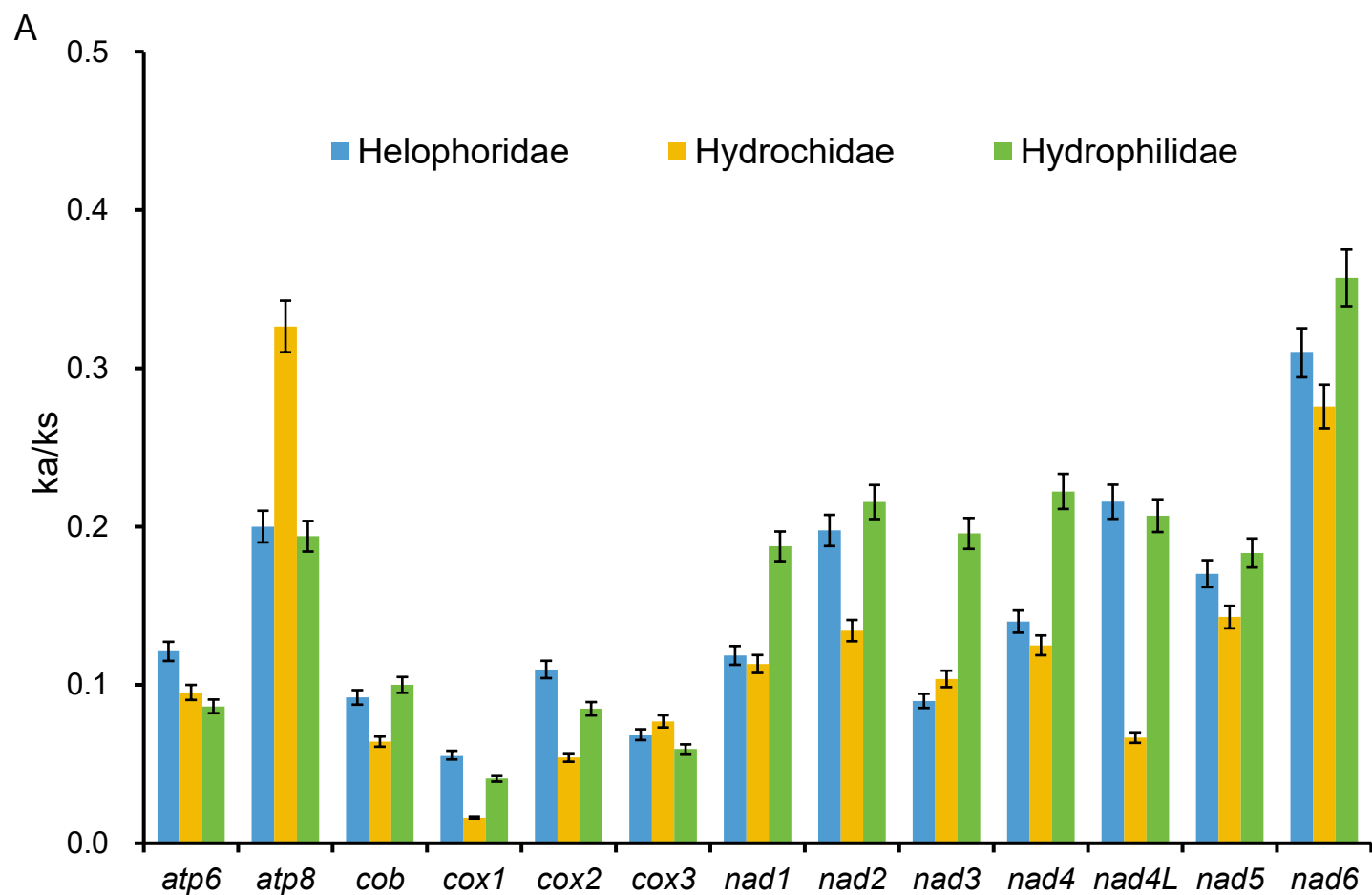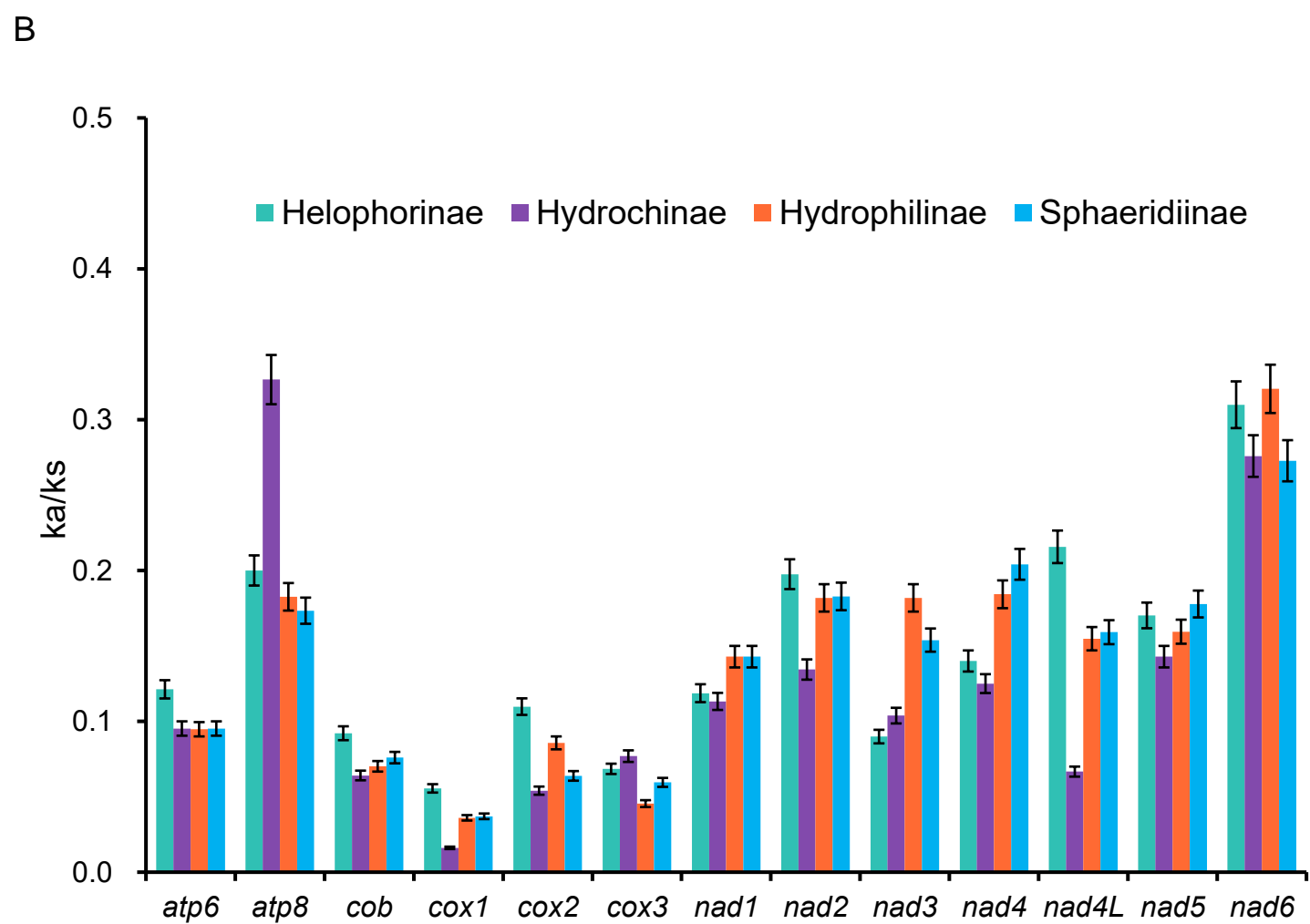

Supplement: Supplementary file 1 [file biology-15-00571-s001.zip › Figure S5 Comparative ω ratio variation across Hydrophiloidea lineages.pdf]

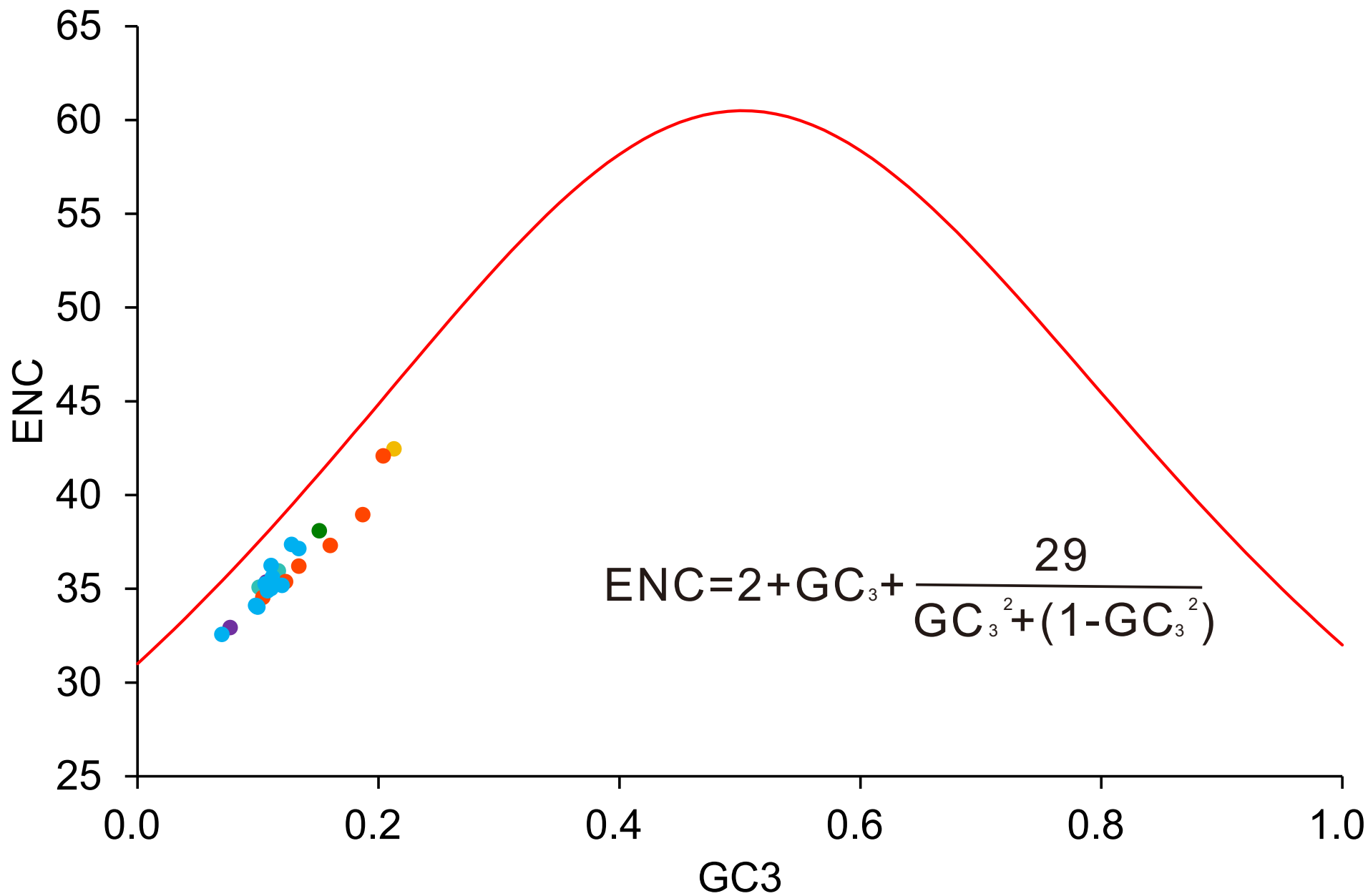

Supplement: Supplementary file 1 [file biology-15-00571-s001.zip › Figure S6 Correlation between ENC values and G+C content.pdf]

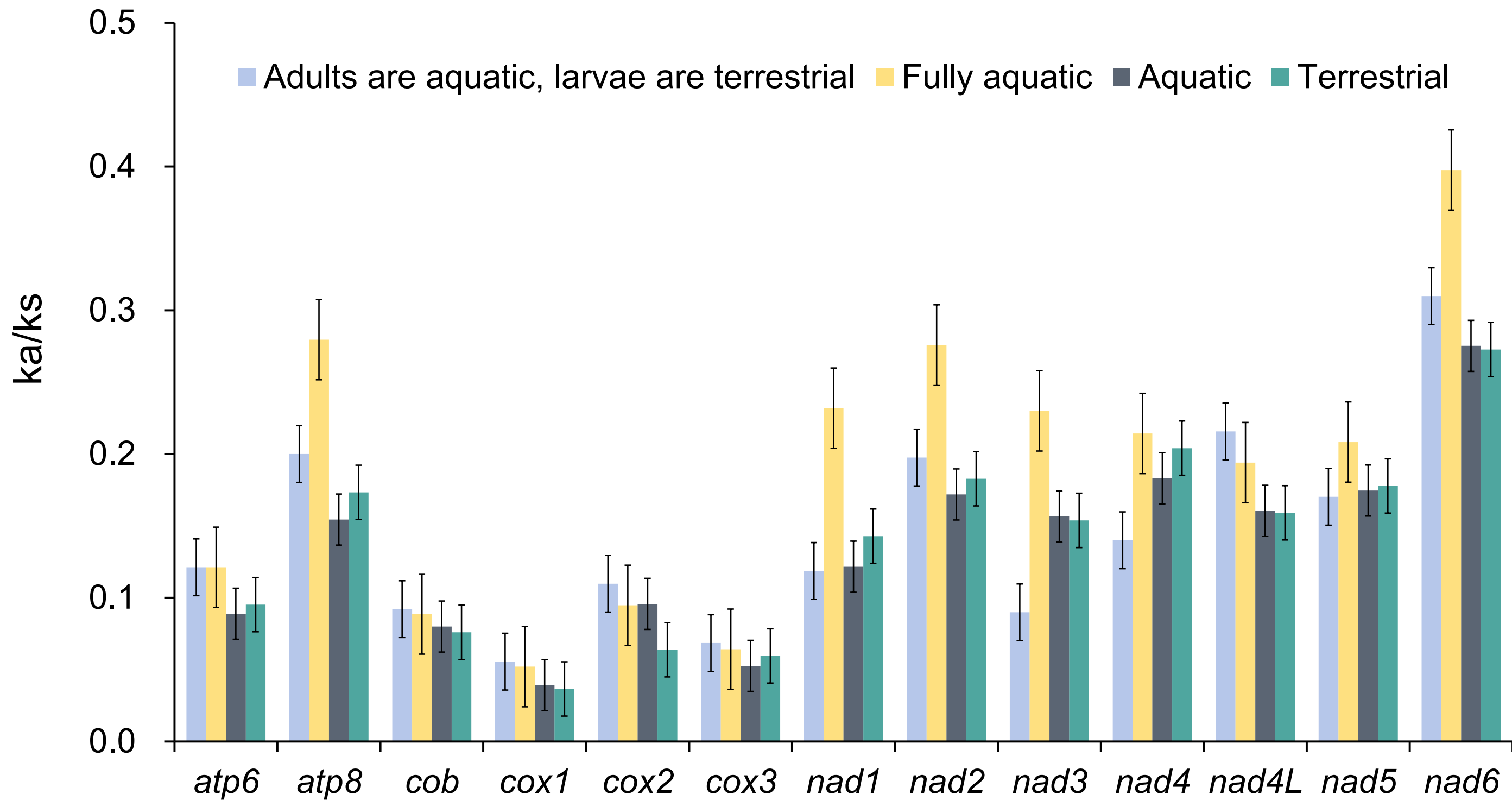

Supplement: Supplementary file 1 [file biology-15-00571-s001.zip › Figure S8 ω variation among Hydrophiloidea species with different habitat types.pdf]
